# Local mRNA translation and cytoskeletal reorganization: Mechanisms that tune neuronal responses

**DOI:** 10.3389/fnmol.2022.949096

**Published:** 2022-08-01

**Authors:** Nikoletta Triantopoulou, Marina Vidaki

**Affiliations:** ^1^Division of Basic Sciences, Medical School, University of Crete, Heraklion, Greece; ^2^Institute of Molecular Biology and Biotechnology, Foundation for Research and Technology Hellas (IMBB-FORTH), Heraklion, Greece

**Keywords:** local mRNA translation, cytoskeleton reorganization, neuronal development, axon elongation and regeneration, synaptogenesis, ribonucleoprotein complexes

## Abstract

Neurons are highly polarized cells with significantly long axonal and dendritic extensions that can reach distances up to hundreds of centimeters away from the cell bodies in higher vertebrates. Their successful formation, maintenance, and proper function highly depend on the coordination of intricate molecular networks that allow axons and dendrites to quickly process information, and respond to a continuous and diverse cascade of environmental stimuli, often without enough time for communication with the soma. Two seemingly unrelated processes, essential for these rapid responses, and thus neuronal homeostasis and plasticity, are local mRNA translation and cytoskeletal reorganization. The axonal cytoskeleton is characterized by high stability and great plasticity; two contradictory attributes that emerge from the powerful cytoskeletal rearrangement dynamics. Cytoskeletal reorganization is crucial during nervous system development and in adulthood, ensuring the establishment of proper neuronal shape and polarity, as well as regulating intracellular transport and synaptic functions. Local mRNA translation is another mechanism with a well-established role in the developing and adult nervous system. It is pivotal for axonal guidance and arborization, synaptic formation, and function and seems to be a key player in processes activated after neuronal damage. Perturbations in the regulatory pathways of local translation and cytoskeletal reorganization contribute to various pathologies with diverse clinical manifestations, ranging from intellectual disabilities (ID) to autism spectrum disorders (ASD) and schizophrenia (SCZ). Despite the fact that both processes are essential for the orchestration of pathways critical for proper axonal and dendritic function, the interplay between them remains elusive. Here we review our current knowledge on the molecular mechanisms and specific interaction networks that regulate and potentially coordinate these interconnected processes.

## Introduction

Neurons are the “foundation stones” of the nervous system (NS), relentlessly processing and conveying information crucial for the orchestration of all the necessary functions of life. They are among the most structurally sophisticated cells, consisting of a cell body (soma) with two molecularly and functionally distinct types of cytoplasmic protrusions: the dendrites and the axon (Kevenaar and Hoogenraad, 2015). The significantly extended axons of higher vertebrates are often required to quickly integrate and process multiple incoming signals, independently of the soma. Thus, it is easy to envisage that the proper development, maintenance, and function of the NS depends on a certain degree of axonal autonomy, as in most cases there is simply not enough time for communication with the soma (Mofatteh, 2020). This semi-autonomous activity of axons strongly relies on two processes: Local mRNA translation and dynamic cytoskeleton reorganization.

Neurons possess free ribosomes in their distal compartments and are thus capable of regulating protein synthesis locally and on-demand, using mRNA molecules that are trafficked to various subcellular locations and maintained in a dormant state unto specific stimulation (Cajigas et al., 2012; Buxbaum et al., 2014; Zappulo et al., 2017; Glock et al., 2021). Since the translation of a single mRNA molecule can generate multiple proteins, localized protein synthesis is considered an energetically favorable mechanism over transport of individual proteins to distal axonal compartments, allowing for drastic alteration of the local proteome and subsequent rapid responses upon receipt of microenvironmental signaling (Gasparski et al., 2022). On the other hand, the neuronal cytoskeleton, composed of actin filaments (filamentous actin or F-actin), neurofilaments, and microtubules (MTs), acts as a key regulator of crucial molecular and cellular events related to the establishment and maintenance of neuronal polarity, morphology, structural integrity, and plasticity (Luo, 2002; Barnes and Polleux, 2009). Even though its name implies a static nature, the cytoskeleton is actually exceptionally dynamic, capable of undergoing rapid rearrangements in order to adjust to emerging cellular needs in response to several stimuli.

Local mRNA translation is highly linked to the cytoskeleton, as the latter not only serves as a platform for mRNA trafficking but also acts as a scaffold for the organization of the translational machinery components (Venticinque et al., 2011; Kevenaar and Hoogenraad, 2015; Piper et al., 2015). Meanwhile, successful cytoskeleton reorganization is accomplished by a cycle of polymerization and depolymerization of filaments, based on monomers that are locally synthesized (Grantham et al., 2002; Buxbaum et al., 2014; Preitner et al., 2014). Despite the obvious interrelation of the two processes, and their well-established roles in the regulation of crucial cellular events during development and adulthood, little is known about the potential molecular mechanisms and complexes that are involved in their coordination. Here, we aim to provide an overview of the current literature on the co-orchestrated regulation of local protein synthesis and cytoskeletal reorganization, both in the developing and adult NS. Our goal is to summarize our knowledge and highlight potential missing information that would help us understand the pivotal interplay between these processes, necessary for proper NS structure and function.

## Importance and regulation of local mRNA translation in neurons

*De novo* protein synthesis localized to specific subcellular regions, a process known as local mRNA translation, can be achieved via targeted mRNA transport and allows for the spatiotemporal control of a cell’s protein repertoire (Holt and Schuman, 2013). Localized protein synthesis has been studied extensively in model organisms, where the specific subcellular localization of cytosolic mRNA molecules (e.g., *bicoid*, *oscar*, *nanos*) is crucial for development and cell fate determination (Forrest and Gavis, 2003; Weil et al., 2008; Zimyanin et al., 2008). For example, due to its large size and polarity, the normal development and function of an oocyte in *Drosophila melanogaster* or *Xenopus laevis*, depends on the targeted transport of translationally silent mRNAs, and the subsequent activation of protein synthesis at a specific developmental stage or location (Besse and Ephrussi, 2008). This differential subcellular localization of mRNAs in different systems of various species serves as an evolutionarily conserved mechanism for the asymmetrical distribution of proteins among each cell compartment (Mofatteh, 2020). Interestingly, the localized mRNAs often times undergo differential cleavage of their untranslated regions (UTRs) that generates compartment-specific protein isoforms (Andreassi et al., 2021). In addition, due to differences in their post-translational modifications (PTMs), newly synthesized proteins resulting from local mRNA translation are also functionally different than their already existing counterparts. For instance, locally synthesized β-actin is critical for cell polarity and directional movement of fibroblasts (Liao et al., 2008).

In the sublimely complex NS, formation of functional neuronal networks necessitates precise axonal and dendritic positioning, which in turn require spatiotemporal regulation of gene expression. Neuronal local translation is an elegant mechanism, key to this regulation; specific mRNAs that are transported in a dormant state in distal neuronal compartments, are translated on-demand, thus providing axons with a level of autonomy that is crucial for their proper development and function (Mofatteh, 2020).

### Local mRNA translation in axon outgrowth and guidance

Several studies highlight the role of local translation in neurodevelopmental processes, such as growth cone formation, axon pathfinding and branching, and formation of synapses, while at the same time numerous neurodevelopmental disorders have been correlated with disrupted local protein synthesis (Sasaki et al., 2010; Zivraj et al., 2010; Spillane et al., 2012; Gkogkas et al., 2013; Santini et al., 2013). After extensive research in the last couple of decades, we now know that developing axons exhibit high capacity for local translation, which provides them with such remarkable autonomy, that they can grow and navigate independently of the neuronal soma. Indeed, a series of elegant *in vitro* experiments initially unraveled that *Xenopus* retinal axon pathfinding remains unaffected by the removal of the soma (Harris et al., 1987). This observation, along with previous pioneer studies that uncovered the presence of ribosomes in the axonal compartment, led to the conclusion that the basic translation machinery is available in axons, allowing them to tune their proteome and respond to their environment as needed (Harris et al., 1987; Mofatteh, 2020). However, the cellular mechanisms that regulate precise positioning of ribonucleoprotein particles (RNPs) in axons remain largely unknown. Recent work in *X. laevis* has shown that axonal mRNAs and RNPs are co-transported with late endosomes, with the latter ones also serving as docks for local translation of proteins responsible for axon survival and integrity (Cioni et al., 2019). Interestingly, mitochondria reside in the endosome hotspots for local translation and numerous of the translated mRNAs correspond to proteins that regulate or maintain mitochondrial functions (Cioni et al., 2019). These observations in good agreement with previous work unraveling the importance of mitochondria in local translation and axon branching downstream of nerve growth factor (NGF) (Spillane et al., 2013), underline a remarkable mechanism that axons display, in order to self-sustain their protein homeostasis and the energy required to do so.

On top of the ability to maintain their integrity, axons need to respond to a multitude of extracellular signals. Especially in the mammalian NS the neuronal microenvironment is rather complex. Axons and their growth cones receive and integrate a plethora of constant chemoattractive and chemorepulsive cues *in vivo*. Successful guidance to their synaptic targets is then achieved via intracellular signal transduction and subsequent directed movement along the correct pathway (Dickson, 2002; Erskine and Herrera, 2007). This directional steering strongly depends on local mRNA translation and several axon guidance molecules and growth factors are responsible for eliciting rapid axonal protein synthesis. For instance, the Slits and Semaphorins serve as extracellular cues that promote local translation of mRNA molecules and induce repulsive turning of axons. Notably, blocking local translation can inhibit repulsive turning in response to these molecules (Piper et al., 2006). On the other hand, attractive cues like Netrin-1 also require local protein synthesis to exert their effect on growing axons. The Netrin-1 receptor DCC has been previously shown to directly interact with the translation machinery in developing axons, thereby promoting translation of specific mRNAs and *de novo* synthesis of proteins necessary for Netrin-1 signaling (Tcherkezian et al., 2021). Notably, one of the best characterized mRNAs that are asymmetrically translated in response to Netrin-1 signaling is that of β*-actin*, thereby providing the growth cone with blocks for rapid cytoskeletal remodeling, and directional steering toward the chemoattractant source (Leung et al., 2006, 2018).

### Local mRNA translation in synapse formation and plasticity

Apart from its evident role in axon development, local protein synthesis is pivotal for synapses. Many studies have shown that the ability of synapses to synthesize proteins in response to specific local demands is necessary for the developing nerve terminal to sense and respond to extrinsic signals. Therefore, it is essential for synaptogenesis, synapse strengthening, and elimination, and even for relaying signals to the cell soma and influencing neuronal survival (Batista and Hengst, 2016). Notably, local translation has been found to be indispensable at both the pre- and post-synaptic sites (Agrawal and Welshhans, 2021). In the post-synaptic compartment, where polyribosomes were first visualized by electron microscopy (EM), a lot of work has been focused on the function of local mRNA translation, which appears as a highly dynamic modulator of the local proteome (Steward and Levy, 1982; Ostroff et al., 2012, 2018). A number of studies highlight a potential similar role of mRNA translation in the pre-synaptic compartment (Koenig, 1967; Autilio et al., 1968; Morgan and Austin, 1968; Koenig et al., 2000; Scarnati et al., 2018). Although initial EM studies were unable to detect ribosomes, or ribosomal RNA (Bartlett and Banker, 1984), recent work has revealed clusters of ribosomes associated with actin in mature axons, as well as a particularly diverse pool of pre-synaptic axonal and dendritic mRNAs (Koenig et al., 2000; Cajigas et al., 2012; Hafner et al., 2019).

A very-well studied signal that induces axon terminal branching and synaptogenesis is the Brain Derived Neurotrophic Factor (BDNF) (Wang et al., 2022). BDNF initiates local translation via activation of tropomyosin receptor kinase B (TrkB) receptors and mTOR signaling, as well as via stimulation of the group I metabotropic glutamate receptors (mGluRs) and ERK-MAPK signaling, regulating synapse formation, plasticity, and maintenance (Steward and Schuman, 2003; Schratt et al., 2004; Napoli et al., 2008). BDNF is secreted both pre- and post-synaptically and affects TrkB receptors localized on both pre- and post-synaptic membranes (Gomes et al., 2006; Mohajerani et al., 2007; Song et al., 2017). Therefore, BDNF activates both pre- and post-synaptic pathways and elicits local protein synthesis and rapid effects on membrane excitability and synaptic transmission (Pradhan et al., 2019). At the pre-synaptic compartment, BDNF-induced activation of TrkB potentiates the release of neurotransmitters such as GABA and glutamate, via the TrkB-ERK pathway (Kang and Schuman, 1995; Levine et al., 1995; Kang et al., 1997; Jovanovic et al., 2000). Post-synaptically, BDNF-induced activation of TrkB generates fast dendritic calcium transients and induces several intracellular signaling pathways (Lang et al., 2007) that may further support structural changes, such as spine density increase and dendritic growth (Segal and Greenberg, 1996; Alonso et al., 2004; Mohajerani et al., 2007). Additional studies have demonstrated that BDNF can induce local synthesis of the transcription factors SMAD1/5/8 followed by their retrograde transport, providing an example of tight signal regulation and relay (Ji and Jaffrey, 2012). In the neuromuscular junction (NMJ) of *D. melanogaster*, a well-investigated invertebrate synaptic system, SMAD proteins are involved in synapse function both in pre- and in post-synaptic cells (Ueberham and Thomas, 2013).

Based on all of the aforementioned observations and a plethora of additional studies, it is unassailable that synaptic plasticity is greatly affected by the levels of local protein synthesis (Biever et al., 2019), and that activity-dependent local translation is essential for the formation and maintenance of long-term memories. For example, it was recently demonstrated that long-term plasticity of GABA release in established synapses requires local translation (Cioni et al., 2018). Concomitantly, an elegant study that perturbed synaptic translation by local depletion of mitochondrial energy compartments, uncovered severe impairment of spine morphological alterations, highlighting the necessity of both protein synthesis and mitochondria during plasticity in hippocampal neurons (Rangaraju et al., 2019).

The significance of local protein synthesis for synapse formation, function, and plasticity is further underlined by the fact that genetic alterations of the pathways that regulate local mRNA translation are associated with the emergence of synaptopathies, the clinical manifestations of which range from mild intellectual disabilities (ID) to autism spectrum disorders (ASD) and schizophrenia (SCZ) (Ehninger and Silva, 2009; De Rubeis et al., 2013). A common anatomical feature of synaptopathies is the dysgenesis of dendritic spines (Penzes et al., 2011; De Rubeis et al., 2013). For instance, Fragile X Syndrome is an inherited synaptopathy characterized by dendritic spine defects which result in neurodevelopmental delays and autistic-like phenotypes (Irwin et al., 2002; Jacquemont et al., 2007; De Rubeis et al., 2013). It is caused by loss of function of FMRP, an RNA-binding protein (RBP) that regulates local mRNA translation and degradation in neurons, and is responsible for the dendritic targeting of mRNAs (Dictenberg et al., 2008; De Rubeis et al., 2013). In addition, defective assembly of RNPs has been associated with the emergence of neurological diseases such as spinal muscular atrophy (SMA) and amyotrophic lateral sclerosis (ALS) (Sanchez et al., 2013; Mofatteh, 2020).

### Local mRNA translation in axon regeneration

Increasing evidence indicates that axonal mRNA translation continues to play roles in mature axons, especially during plastic responses such as injury-induced axon regeneration (Verma et al., 2005; Jung et al., 2012; Kalinski et al., 2015). Interestingly, axonal regeneration following injury encompasses cellular processes that are very similar to physiological axon growth during development, namely axon elongation and the formation of a new growth cone, which is receptive to developmental cues that guide it toward its lost synaptic targets to restore connectivity (Verma et al., 2005; Giger et al., 2010). All these processes require local mRNA translation. And while mature axons of the peripheral nervous system (PNS) maintain the capacity for mRNA trafficking and translation, which is necessary for regeneration after injury, mature axons of the central nervous system (CNS) lose this intrinsic ability (Gumy et al., 2010). This is primarily because the transition from development to maturity in the CNS is marked by gene expression changes that favor synaptic functions and block growth, therefore limiting the capacity of axons for local translation (Jung et al., 2012). *In vitro* as well as *in vivo* studies have shown that the enhancement of protein synthesis in injured axons promotes regeneration, while on the other hand, axonal regeneration is attenuated when mRNA translation is blocked after injury (Verma et al., 2005; Christie et al., 2010; Donnelly et al., 2011; Saxton and Sabatini, 2017). These observations suggest that there is a strong correlation between local mRNA translation and the intrinsic ability of axons to regenerate.

## Structure and functions of the neuronal cytoskeleton

As implied by its name, the main role of the cytoskeleton is not only to provide a structural scaffold, establishing and maintaining the mechanical properties, morphology, polarity, and integrity of neurons but also to contribute to neuronal plasticity (Kevenaar and Hoogenraad, 2015). It is an incredibly dynamic structure, undergoing rapid remodeling in order to meet emerging cellular needs in response to constant environmental stimuli and intrinsic homeostatic processes. Several key cellular and molecular events, including protrusion, motility, macromolecule, and organelle positioning, as well as vesicular trafficking, strongly depend on the cytoskeleton, its dynamic flux and ability to serve as a signaling platform (Kim and Coulombe, 2010).

### Major components of the neuronal cytoskeleton

The neuronal cytoskeleton is built up from three distinct but integrated fibrous polymers: MTs, F-actin, and neurofilaments. MTs are cylindrical polymers comprised of α- and β-tubulin heterodimers, actin filaments are polymers built up from globular actin (G-actin) and neurofilaments are a family of neuronal intermediate filaments (Kevenaar and Hoogenraad, 2015). F-actin and MTs can serve as rails for long- and short-range axonal transport and can influence axon growth and specification (Kevenaar and Hoogenraad, 2015). Neurofilaments are found mostly in axons and serve as regulators of their diameter and conductance (Yuan et al., 2017).

MTs are characterized by their highly dynamic nature, and their continuous growth and shrinkage constitute the main driving forces for rapid cytoskeletal remodeling (Kevenaar and Hoogenraad, 2015). The filaments can exist in a stable state, marked by PTMs, or they can be dynamically unstable, stochastically switching between polymerization and depolymerization (Mitchison and Kirschner, 1984; Janke, 2014). This is regulated by a wide array of factors, among which are the microtubule-associated proteins (MAPs), with various MAPs, such as MAP1B and Tau, influencing MT dynamics by inducing their stabilization (Drechsel et al., 1992; Tortosa et al., 2013; Derisbourg et al., 2015). One major characteristic of axonal MTs in particular is their unipolar organization, with their plus-end (fast-growing) oriented toward the axon tip and the minus-end (more stable) located in the opposite direction, toward the soma (Baas et al., 1988; Stepanova et al., 2003; Stone et al., 2008). Interestingly though, MTs are excluded from dendritic spines and can only be found in dendritic shafts with mixed orientation (Baas et al., 1988; Stone et al., 2008; Kapitein et al., 2010).

F-actin is another actively dynamic structure, rapidly switching between polymerization and depolymerization, due to the weak interaction forces of actin monomers. Actin monomers are added to the growing end of the protrusion, while actin subunits dissociate in the opposite end (Letourneau, 2009). They are also highly polarized due to the orientation of the actin monomers in the filament (Kevenaar and Hoogenraad, 2015). Many actin-binding proteins (ABPs) influence actin dynamics through different mechanisms, such as the promotion of polymerization/depolymerization of G-actin, as well as the crosslinking and anchoring of F-actin to other cellular components (Letourneau, 2009). Axonal actin is organized along the axon in ring-like structures, comprised of short actin filaments connected by spectrin tetramers and caped by adducin (Xu et al., 2013; Lukinavièius et al., 2014; D’Este et al., 2015). Concomitantly, the cytoskeleton of the dendritic spines is composed of a highly branched network of long- and short-branched actin filaments, connected by many ABPs (Nakahata and Yasuda, 2018). Rearrangements of the actin cytoskeleton, such as actin polymerization/depolymerization, branching, cross-linking, and trafficking, are regulated by multiple ABPs and small GTPase proteins and influence the formation, shape, motility, and stability of dendritic spines (Harvey et al., 2008; Murakoshi et al., 2011; Bosch et al., 2014; Hedrick et al., 2016).

### The cytoskeleton during axon outgrowth and guidance

It has become clear that the cytoskeleton enacts a central role during neuronal development, acting as a signaling platform and generating intracellular forces which regulate the speed and direction of outgrowth (Tanaka and Sabry, 1995). Localized cyclic polymerization and depolymerization of F-actin, in combination with MTs stabilization, and chaperoning events like Kinesin1-mediated sliding of MTs contribute to the generation of the mechanical forces needed for the induction of neurite outgrowth (Flynn et al., 2012). The initial exploration stage is characterized by the rapid multidirectional extension and retraction of actin protrusions in response to microenvironmental cues (Tanaka and Sabry, 1995). In particular, in the growth cones that are the distal tips of growing axons, highly dynamic actin filaments that originate from a meshed actin network in the leading edge (lamellipodium), generate filopodial protrusions that serve as cellular antennae, sampling the microenvironment for numerous attractive and repellent extracellular signals (Kolodkin and Tessier-Lavigne, 2011). This plethora of external cues promotes the activation of intracellular cascades, which ultimately converge on the cytoskeleton and induce local rearrangements that contribute to the overall neuronal response and guidance toward the correct direction (Vitriol and Zheng, 2012). Following actin protrusions and signaling, the MTs explore the new growth cone intracellular environment, in order to stabilize the navigating axons and promote proper directionality (Pinto-Costa et al., 2020). Signal transduction in the growth cones depends on factors such as the Rho subfamily of Ras-related GTPases (Tanaka and Sabry, 1995), while numerous ABPs such as Cofilin 1 (Cfl1), Profilins, and Ena/VASP family members are essential for actin reorganization (Dent et al., 2011).

### The cytoskeleton during synapse formation and plasticity

Upon reaching the appropriate synaptic target, axonal growth cones cease to explore the environment and axons attenuate their growth, initiating the formation of branches and ultimately presynaptic sites, with crucial cytoskeletal rearrangements (Kevenaar and Hoogenraad, 2015). Specifically, NGF-mediated localized debundling of MTs promotes the formation of axon branches, whereas actin assembly contributes to the stabilization of the branch (Ketschek et al., 2015). Cytoskeletal rearrangements are also pivotal in dendritic spines, although the proper function of neuronal circuits strongly depends on the capacity of spines to remain in a stable state for long periods of time. Numerous studies point to the significance of dynamic molecular and subsequent structural reorganization of spines for circuit plasticity during learning (Grutzendler et al., 2002; Trachtenberg et al., 2002; Yang et al., 2009; Hayashi-Takagi et al., 2015; Li et al., 2017). Synaptic activity regulates dendritic spine morphology by influencing the reorganization of MTs and F-actin in both the developing and adult NS (Gordon-Weeks and Fournier, 2014). The balance between polymerization and depolymerization of actin networks is essential in synaptic plasticity, inducing activity-dependent structural alterations in dendritic spines (Hotulainen and Hoogenraad, 2010). On the other hand, MTs in the dendritic shafts also undergo rapid structural changes that are necessary for spine plasticity and maintenance of proper spine structure (Jaworski et al., 2009; Merriam et al., 2011, 2013). On top of their structural role though, both actin and microtubule filaments have been shown to be of utmost importance for the tethering and stabilization of dendritic mitochondrial compartments, which provide spines with the fuel required for synaptic local translation and plastic responses (Rangaraju et al., 2019).

### The cytoskeleton during axon regeneration

Successful axon regeneration, following trauma induced by mechanical injury or a neurodegenerative disease, strongly relies on the capacity of the cytoskeleton for rapid and coordinated rearrangements (Kevenaar and Hoogenraad, 2015). One of the reasons why adult CNS axons fail to regenerate after injury is the inhibitory microenvironmental cues that ultimately suppress cytoskeletal reorganizations, required for axon regrowth (Gordon-Weeks and Fournier, 2014). Upon CNS injury, the retraction bulbs that are formed at the tips of the injured neurons are dystrophic and growth-incompetent, comprised of a severely disorganized cytoskeleton (Ertürk et al., 2007). Conversely, the bulbs that are formed at the tips of injured PNS neurons consist of a highly organized and appropriately bundled network of MTs and dynamic actin structures, which resemble a developmental growth cone and can promote axon regrowth and regeneration (Gordon-Weeks and Fournier, 2014). Inhibitory molecules converge upon the neuronal cytoskeleton by initiating intracellular signaling cascades that hinder axonal regrowth. The small GTPase RhoA, as well as its downstream effector Rho Kinase (ROCK), are central mediators of the actin cytoskeleton, regulating inhibitory signaling pathways that limit regeneration (Alabed et al., 2006; McKerracher and Higuchi, 2006). Various inhibitory ligands activate RhoA-mediated cascades, therefore blocking axonal repair. Several studies have indicated that RhoA or ROCK inhibition improves axonal regeneration by enhancing the *in vitro* and *in vivo* regrowth of injured CNS neurons on repellent substrates (Wahl et al., 2000; Dergham et al., 2002; Borisoff et al., 2003; Fournier et al., 2003; Yukawa et al., 2005; Alabed et al., 2006). Therefore, RhoA inhibitors display therapeutic potential and are currently used in clinical trials aiming to enhance axonal regeneration after spinal cord injury (Fehlings et al., 2011; Pinto-Costa et al., 2020).

### The cytoskeleton in transport

Besides its crucial aforementioned roles, the cytoskeleton is an essential element for the active transport of a plethora of molecular cargoes along the significantly extended projections of neurons. Neuronal homeostasis, axonal polarization and outgrowth, synaptic function, and regeneration of axons after injury are all processes that rely on cytoskeleton-dependent transport. Conserved mechanisms of active motor protein-mediated transport are crucial for the proper distribution of various types of cargoes, such as axonal proteins, mRNAs, signaling molecules, vesicles, and organelles, at specific locations within the axons and dendrites (Broix et al., 2021). Three types of motor proteins mediate the cytoskeletal transport of the aforementioned cargoes: Myosins, kinesins, and dyneins. Kinesin is responsible for the anterograde long-range transport (from the soma to the synapse) of cargoes along the MTs, while dynein is involved in the retrograde long-range transport (from the synapse to the soma) (Hirokawa et al., 2010). In contrast to the previous motor proteins, myosin moves along the actin filaments of the cytoskeleton and facilitates short-range transport (Vale, 2003). Notably, dynein-dependent retrograde axonal transport is one of the first cellular processes activated after NS injury, in order for neurons to induce a regenerative response (Broix et al., 2021).

## Coordination between local translation and cytoskeletal remodeling

It is more than evident that cytoskeletal dynamics are crucial for proper architecture, function, and regeneration of the NS, and indeed, numerous studies have linked cytoskeletal defects to the emergence of neurodevelopmental and neurodegenerative diseases (Kevenaar and Hoogenraad, 2015). Concomitantly, local protein synthesis is pivotal for neuronal homeostasis, with an increasing number of studies unraveling its role during development, plasticity, and regeneration. The two processes are essentially interrelated and it has been recently demonstrated that cytoskeletally-tethered mitochondria exist in dendrites, as local energy booths that fuel local translation in synapses and potentially other neuronal compartments (Rangaraju et al., 2019). However, what remains poorly understood up to date, are the molecular mechanisms neurons utilize to co-regulate local protein synthesis and cytoskeletal remodeling, when rapidly responding to stimuli.

### The CYFIP1 complexes

#### The FMRP-CYFIP1 ribonucleoprotein complex

One of the best-characterized mechanisms that coordinate cytoskeletal remodeling and local translation involves the FMRP-CYFIP1 RNP complex. The cytoplasmic fragile X mental retardation protein (FMRP) interacts with the Cytoplasmic FMRP Interacting Protein (CYFIP1), also known as Specific Rac1-Activated protein (SRA1), forming a heterodimer ribonucleoparticle that represses protein synthesis (Kobayashi et al., 1998; Schenck et al., 2001, 2003; Napoli et al., 2008). FMRP is an RBP implicated in mRNA translation, localization, and stability (Bagni and Greenough, 2005; Zalfa et al., 2007; Zhang et al., 2007). It is the encoded protein product of the X-linked fragile X mental retardation 1 (*fmr1*) gene. FMRP interacts with specific mRNA molecules by recognizing domains such as G quartets and/or U-rich sequences, or via small non-coding RNA adaptors and miRNAs (Napoli et al., 2008). It influences the dendritic targeting of mRNAs and regulates mRNA translation and decay in the neuronal soma and at synapses (Bassell and Warren, 2008; Dictenberg et al., 2008). The other crucial partner of the FMRP-CYFIP1 RNP complex is CYFIP1, a protein that regulates both cytoskeletal dynamics and protein translation. FMRP tethers specific mRNAs to CYFIP1, which in turn interacts and binds to the cap-binding eukaryotic initiation factor 4E (eIF4E) and inhibits the initiation of translation (Napoli et al., 2008; De Rubeis et al., 2013). Extracellular cues, like BDNF, and synaptic activity result in the release of CYFIP1 from eIF4E and from bound mRNAs, promoting the initiation of mRNA translation (Napoli et al., 2008). Protein synthesis can then begin after the eukaryotic initiation factor 4G (eIF4G) binds to eIF4E, promoting the recruitment of other initiation factors and ribosomal proteins (Santini et al., 2017). Several mRNAs have been identified to be translationally inhibited by the FMRP-CYFIP1 complex in the mammalian brain, including *map1b*, *camkII*, *arc*, and *app*. Indeed, the absence of any of the two, CYFIP1 or FMRP, has been associated with increased translation levels of the aforementioned mRNAs (Zhang et al., 2001; Zalfa et al., 2003; Lu et al., 2004; Hou et al., 2006; Westmark and Malter, 2007; Napoli et al., 2008). In dendrites and synapses, BDNF promotes the FMRP-CYFIP1-mediated translation of *arc/arg3.1* and *camkII* (Aakalu et al., 2001; Yin et al., 2002; Zalfa et al., 2003; Schratt et al., 2004; Napoli et al., 2008).

#### The CYFIP1-WRC complex

Besides the FMRP-CYFIP1-eIF4E complex, CYFIP1 has also been identified as part of the WAVE Regulatory Complex (WRC). WRC is implicated in actin polymerization by regulating the actin-nucleating activity of the Arp2/3 complex (Schenck et al., 2001; Kunda et al., 2003; Napoli et al., 2008; De Rubeis et al., 2013). It is a heteropentamer, containing WAVE1/2/3, ABI1/2, NCKAP1, and HPSC300, and can be activated through kinases and phospholipids, as well as through the small Rho GTPase Rac1, which induces a CYFIP1-mediated activation of WRC (Kobayashi et al., 1998; Eden et al., 2002; Takenawa and Suetsugu, 2007; Chen et al., 2010).

Both CYFIP1 complexes are crucial for proper synaptic function, since they establish a fine balance between cytoskeletal reorganization and mRNA translation. The incorporation of CYFIP1 in each complex relies on the capacity of CYFIP1 to undergo conformational changes. Specifically, a more globular CYFIP1 conformation is required for the assembly of the FMRP-CYFIP1-eIF4E complex, while a planar form is suitable for the recruitment of CYFIP1 to the WRC (Chen et al., 2010; De Rubeis et al., 2013). This conformational change of CYFIP1 is promoted by factors such as BDNF. BDNF administration results in a Rac1 signaling-mediated conformational transition of CYFIP1 from globular to planar (De Rubeis et al., 2013). This reduces the amount of CYFIP1 interacting with FMRP and, as a result, enhances protein synthesis of key regulators of synaptic plasticity, such as ARC (De Rubeis et al., 2013). Concomitantly, it increases the pool of CYFIP1 recruited on the WRC, promoting actin cytoskeleton rearrangements, necessary for proper spine morphology and function (De Rubeis et al., 2013).

Perturbations in the balance of these two CYFIP1 interconnected pathways are associated with spine dysmorphogenesis, a recurrent feature of several neuropsychiatric disorders (Penzes et al., 2011; De Rubeis et al., 2013). In particular, loss of function of FMRP causes the Fragile X Syndrome (FXS), a common inherited ID, also implicated in the emergence of ASD (Hatton et al., 2006; Jacquemont et al., 2007; Bassell and Warren, 2008). At the cellular level, FXS is characterized by deficient synaptic maturation, while patients with FXS display dendritic spine defects, autistic-like phenotypes, and neurodevelopmental delays (Irwin et al., 2002; Jacquemont et al., 2007). A model proposed by Napoli et al. (2008) suggests that in the absence of FMRP, there would be decreased binding of CYFIP1 to FMRP target mRNAs and subsequent alleviation of translational inhibition. This would result in abnormally high levels of proteins whose synthesis undergoes FMRP regulation (Napoli et al., 2008). Since a wide array of mRNAs is regulated by FMRP, the simultaneous dysregulation of numerous proteins may contribute to the emergence of FXS (Brown et al., 2001; Miyashiro et al., 2003; Liao et al., 2008; Darnell et al., 2011; Klemmer et al., 2011).

*cyfip1* is located at the 15q11.2 chromosomal locus, a hot-spot for ASD. Mutations that lead to downregulation of *cyfip1* mRNA levels have been associated with cognitive disabilities and ASD (Doornbos et al., 2009; van der Zwaag et al., 2010; Cooper et al., 2011; von der Lippe et al., 2011). In addition, downregulation of the *cyfip1* mRNA has been observed in a subgroup of FXS patients who display a Prader-Willi-like phenotype, severe ASD, and obsessive-compulsive behavior (Nowicki et al., 2007; De Rubeis et al., 2013). CYFIP1 has also been linked to SCZ (Tam et al., 2010; Zhao et al., 2013). Depletion of CYFIP1 negatively influences ARC synthesis and actin polymerization, severely affecting spine morphology (De Rubeis et al., 2013). Not only CYFIP1 but also a plethora of CYFIP1 interactors, among which NCKAP1 and eIF4E, are implicated in disorders with a broad range of clinical manifestations, such as ID, ASD, and SCZ (Nowicki et al., 2007; Doornbos et al., 2009; Neves-Pereira et al., 2009; Tam et al., 2010; Cooper et al., 2011; Zhao et al., 2013).

Genetic ablation of the WRC components is also associated with defective rearrangements of the actin cytoskeleton, which negatively influence dendritic spine homeostasis, morphology, and excitability (Grove et al., 2004; Wiens et al., 2005; Kim et al., 2006; Soderling et al., 2007). Regarding the potential therapeutic strategies for these synaptopathies, Santini et al. (2017) proposed an interesting viewpoint related to the treatment of FXS. Particularly, they showed that treating FXS mice with 4EGI-1, which blocks interactions between eIF4E and eIF4G that are required for protein synthesis, reverses defects in hippocampus-dependent memory and spine morphology (Santini et al., 2017). Since the aberrant increase in the levels of many proteins is associated with the emergence of FXS, the targeting of translation initiation factors may be a promising therapeutic plan.

### The mena-ribonucleoprotein complex

Another important player involved in cytoskeletal dynamics and local translation is the Enabled/Vasodilator-Stimulated Phosphoprotein (Ena/VASP) family. Three Ena/VASP family members are found in vertebrates: Mena (Mammalian-Enabled), VASP (Vasodilator-Stimulated Phosphoprotein), and EVL (Ena-VASP like). The Ena/VASP proteins contain two conserved domains: the N-terminus EVH1 (Ena-VASP Homology 1) and the C-terminus EVH2. The EVH1 domain binds to proteins with FPPPP (FP4) repeats and is crucial for cellular localization (Bilancia et al., 2014; Harker et al., 2019). EVH2 is composed of an F-actin binding domain (FAB), a G-actin binding domain (GAB), and a C-terminal coiled-coil tetramerization domain (Bachmann et al., 1999; Walders-Harbeck et al., 2002). Between EVH1 and EVH2, there is a central poly-proline region that binds the monomer-binding protein profilin 1 (PFN1) (Ferron et al., 2007; Hansen and Mullins, 2010; Harker et al., 2019), which is necessary for both Arp2/3 and Ena/VASP function (Skruber et al., 2020). All Ena/VASP members display actin filament anti-capping and barbed-end elongation enhancement activity (Barzik et al., 2005; Hansen and Mullins, 2010; Breitsprecher et al., 2011; Winkelman et al., 2014; Havrylenko et al., 2015), which renders them crucial for lamellipodia-based motility and the assembly of filopodia (Grevengoed and Peifer, 2003; Gates et al., 2007; Kwiatkowski et al., 2007; Tucker et al., 2011; Havrylenko et al., 2015). At the initial stage of filopodia assembly, Ena/VASP proteins localize at the edge of the lamellipodia protrusions and facilitate the formation of straight, long actin filaments (Bear et al., 2002; Svitkina et al., 2003; Barzik et al., 2005; Applewhite et al., 2007; Bear and Gertler, 2009; Winkelman et al., 2014; Harker et al., 2019). Their localization at the tips of newly formed and mature filopodia promotes the subsequent assembly of fascin-bundled filaments of the same length (Svitkina et al., 2003; Winkelman et al., 2014; Harker et al., 2019). The capacity of Ena/VASP proteins to bind G-actin, F-actin, and profilin and, hence, deliver monomers from the actin-binding sites to the growing barbed ends of actin filaments, is crucial during these processes and enhances motility and protrusion (Chereau and Dominguez, 2006; Ferron et al., 2007; Hansen and Mullins, 2010; Breitsprecher et al., 2011).

Given their pivotal roles in actin-remodeling, Ena/VASP proteins are key players during cell movement and adhesion. Especially in the NS, numerous genetic studies have shown that the Ena/VASP family members are critical factors for neurulation, neuritogenesis, migration, axon guidance and branching, and synapse formation (Lanier et al., 1999; Lebrand et al., 2004; Menzies et al., 2004; Li et al., 2005; Dwivedy et al., 2007; Kwiatkowski et al., 2007; Lin et al., 2007; McConnell et al., 2016). Neurons deficient for Ena/VASP proteins fail to respond to axon guidance cues that elicit both local translation and actin reorganization in axons, such as Netrin and Slit (Lanier et al., 1999; Lebrand et al., 2004; Menzies et al., 2004; McConnell et al., 2016).

Regarding the role of the Ena/VASP family in local mRNA translation, recent findings point to a Mena-dependent regulation. Mena (ENAH), being an actin-regulatory protein, has been implicated in integrin-mediated signaling, cell motility, and adhesion in the developing and adult NS (Drees and Gertler, 2008; Gupton and Gertler, 2010; Gupton et al., 2012). A recent study by Vidaki et al. (2017) revealed an additional role of Mena as a regulator of both steady-state and BDNF-induced local translation in axons. Mena was found to associate with multiple RBPs and is a main component of a novel RNP complex involved in localized mRNA translation in axons (Vidaki et al., 2017). This complex contains known regulators of translation, like HnrnpK, Pcbp1, and additional RBPs, as well as specific cytosolic mRNAs involved in NS development and function, such as *dyrk1a* (Vidaki et al., 2017). Notably, Dyrk1a is a dosage-sensitive, dual-specificity kinase important in neuronal development and implicated in the emergence of ASD, ID, Down syndrome, and Parkinson’s disease (Tejedor and Hämmerle, 2011; O’Roak et al., 2012; Qian et al., 2013; Krumm et al., 2014; Coutadeur et al., 2015; Di Vona et al., 2015; van Bon et al., 2016). Although the localization, and thus axonal transport of *dyrk1a* mRNA is not affected in the absence of Mena, translation of the mRNA is Mena-dependent, and the study revealed a significant decrease in Dyrk1a protein levels, both locally in axons, and globally in Mena-null developing brains (Vidaki et al., 2017). HnrnpK and PCBP1 can form complexes that bind to the 3′-UTRs of target mRNAs, inhibiting the initiation of translation, therefore Mena could be required for the disassembly of the RNP and de-repression of translation (Gebauer and Hentze, 2004; Vidaki et al., 2017). This hypothesis, combined with the facts that both Mena and HnrnpK are implicated in synapse formation and plasticity and that Mena-deficient mice exhibit severe axon guidance defects, highlights the significance of Mena in NS formation and function. Mena’s capability of binding to different growth cone receptors, and its dual role in regulating actin rearrangements and local proteins synthesis, could act as a balancing force between the two processes, coupling and coordinating them on spatiotemporal demand (Lanier et al., 1999; Giesemann et al., 2003; Li et al., 2005; Folci et al., 2014; McConnell et al., 2016; Vidaki et al., 2017).

### The DCC cell-surface receptor

The DCC (Deleted in colorectal cancer) receptor is another example of a molecule that could coordinate local mRNA translation and cytoskeletal reorganization, downstream of Netrin-1 signaling. DCC is a ∼185 kD protein encoded by the *DCC* gene, which is located on chromosome 18q (Keino-Masu et al., 1996). It is a single-pass transmembrane receptor for the extracellular factor Netrin-1, facilitating important functions related to axonal and dendritic growth, guidance, and targeting during development (Keino-Masu et al., 1996; Fazeli et al., 1997; Parent et al., 2005; Tcherkezian et al., 2021). Its extracellular portion contains six fibronectin type III (FN1-FN6) domains and four immunoglobulin-like domains, whereas its intracellular part is comprised of three domains, P1, P2, and P3 (Kolodziej et al., 1996; Finci et al., 2017). DCC can be found in various neuronal populations, expressed across the lifespan of many species, including humans, but its levels decrease dramatically following the transition from embryonic life to adulthood (Manitt et al., 2011; Horn et al., 2013; Reyes et al., 2013; Torres-Berrio et al., 2020). This decrease is accompanied by a change in the role of DCC-mediated signaling, which henceforth is crucial for neuronal survival, and the organization and refinement of large neuronal circuits (Torres-Berrio et al., 2020). Netrin-1, a member of the laminin superfamily, is a secreted protein that binds to the FN4 and FN5 domains of DCC and promotes local protein synthesis and reorganization of the actin cytoskeleton (Torres-Berrio et al., 2020; Tcherkezian et al., 2021). Upon Netrin-1 binding, DCC serves as a platform for the assembly of a multicomponent complex, where numerous intracellular components associated with the translation initiation machinery are recruited (Torres-Berrio et al., 2020; Tcherkezian et al., 2021). Regulation of local translation is mediated by factors such as the Nck-1 adaptor protein and the ribosomal protein L5, which link DCC to the large and small ribosomal subunits (Tcherkezian et al., 2021). Nck-1 activates Src family kinases and Rho GTPases, enhancing the release of Ca^2+^ and initiating local translation and actin cytoskeleton rearrangements (Torres-Berrio et al., 2020). The cytoplasmic domain of DCC also interacts with eukaryotic initiation factors (eIFs) such as eIF4E, which facilitates the recruitment of mRNAs in the preinitiation translation complex, 80S ribosomes, ribosomal subunits 40S and 60S, and various signal transduction proteins implicated in translational control (Tcherkezian et al., 2021). In parallel, Netrin-1-binding to DCC activates PKA that leads to Ena/VASP-dependent actin polymerization, and initiates a signaling cascade that results in Wave-Arp2/3-dependent actin filament branching (Lebrand et al., 2004; Bouchard et al., 2008; Boyer and Gupton, 2018). Therefore, DCC poses as a compelling molecule that coordinates cytoskeletal remodeling and local mRNA translation downstream of Netrin-1, leading to tightly regulated neuronal responses.

Additional guidance receptors, like Robo2/3 that binds Slit2 and Nrp1 that binds Sema3A, elicit local translation during axon development, as well as cytoskeletal reorganization (Piper et al., 2006; Leung et al., 2013; Bellon et al., 2017; Russell and Bashaw, 2018). However, their mechanism of function is either indirect, or elusive, with respect to the immediate coordination of the two processes, and thus they will not be discussed further.

### The APC-ribonucleoprotein complex

An additional molecular network that appears to co-regulate and interrelate local protein synthesis and MT filaments, is the APC-RNP complex. Adenomatus Polyposis Coli (APC) was initially identified as a tumor suppressor, mutated in numerous human colon carcinomas and brain tumors (Powell et al., 1992; Sieber et al., 2002; Green and Kaplan, 2003; Kawasaki et al., 2003; Attard et al., 2007). Structurally, APC is a large scaffold protein with binding domains for several protein targets (Preitner et al., 2014). It is also an MT plus-end tracking protein (+ TIP), involved in the regulation of MT dynamics, playing important roles in cell polarity, adhesion, axon migration, and regulation of the cytoskeleteon (Shi et al., 2004; Watanabe et al., 2004; Etienne-Manneville et al., 2005; Kita et al., 2006; Koester et al., 2007; Purro et al., 2008). In migrating cells, APC has been observed at the ends of detyrosinated MTs (Glu-MTs), where it associates with a minority of MTs toward the leading edge of growing cellular protrusions, promoting MT assembly (Näthke et al., 1996; Mimori-Kiyosue et al., 2000; Wen et al., 2004; Mili et al., 2008).

Apart from regulating MT dynamics, APC associates with both mRNAs and RBPs and forms APC-RNPs involved in local mRNA translation (Mili et al., 2008; Preitner et al., 2014). A genome-wide study by Mili et al. (2008) in migrating fibroblasts revealed a function of APC in RNA localization, as well as a novel RNA anchoring mechanism. They specifically proposed that APC is a component of RNP complexes that contain localized RNAs, *pabp1* and *fmrp*, and is required for accumulation and anchoring of mRNA transcripts in pseudopodial protrusions (Mili et al., 2008). These transcripts are anchored in granules located at the plus ends of Glu-MTs via their 3′-UTRs (Mili et al., 2008). Another genome-wide study by Preitner et al. (2014) in native brain tissue identified APC as an RBP, which serves as a binding platform for a wide array of functionally related protein and mRNA targets. Among these molecular targets are β*-catenin*,β*-actin*, and *importin-*β, which are known to be locally translated in axons and in the leading edge of migrating cells, as well as β*2B-tubulin* (Hanz et al., 2003; Condeelis and Singer, 2005; Jones et al., 2008; Preitner et al., 2014). β2B-tubulin (Tubb2b) is a tubulin isotype implicated in cortical neuron migration and axon tract formation in humans (Jaglin et al., 2009; Cederquist et al., 2012; Romaniello et al., 2012; Preitner et al., 2014). Preitner et al. (2014) suggested a model where APC can induce MT polymerization partially by directing the local protein synthesis of β2B-tubulin in the periphery of MT growing ends. To do so, APC initally binds to the 3′-UTR of β*2B-tubulin* mRNA in order to facilitate its translocation to the dynamic MTs, located in the axonal growth cone’s periphery (Preitner et al., 2014). Subsequently, APC acts as a positive regulator of local translation, promoting β2B-tubulin protein synthesis (Preitner et al., 2014). This axonal enrichment of β2B-tubulin protein at the periphery influences MT dynamics by promoting their further extension and thus contributing to the formation of the axonal growth cone’s expanded structure (Preitner et al., 2014). APC functions have also been implicated in the canonical Wnt/β-catenin signaling pathway, which is known to regulate gene transcription (Rubinfeld et al., 1993; Zeng et al., 2005; Clevers and Nusse, 2012). In accordance with this fact, Preitner et al. (2014) showed that APC binds β*-catenin* mRNA, as well as the mRNAs of several other proteins involved in Wnt/β-catenin signaling, supporting the notion that APC might significantly influence this specific pathway. An additional study from Yasuda et al. (2013) further specifies the way APC mediates local translation. The group showed that Fus is a component of APC-RNPs that preferentially affects protein synthesis within cellular protrusions, and they specifically revealed that local protein synthesis from APC-RNPs can take place within cytoplasmic Fus granules (Yasuda et al., 2013).

In accordance with its roles in MT dynamics and translation, and therefore in numerous aspects of neuronal cell biology, disruption or loss of APC function has been associated with impaired polarization and cell migration, and has also been implicated in neurological disorders, such as SCZ and autism (Etienne-Manneville and Hall, 2003; Watanabe et al., 2004; Kroboth et al., 2007; Kalkman, 2012).

### Zipcode binding protein

Zipcode binding protein (ZBP1) is an oncofetal protein that belongs to a family of highly conserved RBPs and is crucial for proper NS development (Nicastro et al., 2017). Three paralogs are found in vertebrates: IMP1/ZBP1, IMP2, and IMP3/VgRBP (Yisraeli, 2005). In neurons, ZBP1 and VgRBP are localized in growth cones and associate with β*-actin* transcripts (Zhang et al., 2001; Leung et al., 2006; Yao et al., 2006; Welshhans and Bassell, 2011). ZBP1 contains four hnRNP K-homology domains and two RNA recognition motifs (Nielsen et al., 1999; Yisraeli, 2005). Normally, ZBP1 is highly expressed in embryos, and reduced levels, or impaired protein function hinders embryonic development and results in a smaller cerebral cortex (Nishino et al., 2013). In developing neurons, ZBP1 regulates dendritic morphology, growth cone guidance, and axonal remodeling (Eom et al., 2003; Leung et al., 2006; Sasaki et al., 2010; Welshhans and Bassell, 2011; Medioni et al., 2014). To do so, ZBP1 interacts with a wide array of mRNAs (Jønson et al., 2007; Hansen and Mullins, 2010; Patel et al., 2012; Conway et al., 2016; Hafner et al., 2019). This interaction is required for translational regulation, transport, and maintenance of mRNAs, with the most-studied one being that of β*-actin* (Leeds et al., 1997; Hüttelmaier et al., 2005; Leung et al., 2006; Weidensdorfer et al., 2009; Conway et al., 2016). Despite the fact that ZBP1 does not directly associate with the cytoskeleton, its role in the precise localization and translation of β-actin renders it worth mentioning herein.

β-actin is a crucial factor during neuronal development, favoring actin polymerization, cellular remodeling, and migration (Jung et al., 2014; Nicastro et al., 2017). For instance, in the growth cones of developing axons, local β-actin synthesis is essential in steering (Welshhans and Bassell, 2011). Localization of β*-actin* mRNA to subcellular sites of actin polymerization requires ZBP1 (Lawrence and Singer, 1986). ZBP1 binds to “zipcode,” a conserved 54-nucleotide element in the 3′-UTR of the β*-actin* mRNA and facilitates its translocation to actin-rich protrusions, such as the developing neuronal growth cone (Farina et al., 2003; Hüttelmaier et al., 2005). A study of Hüttelmaier et al. (2005) in NG108-15 neuroblastoma cells describes the best characterized mechanism of ZBP1-regulated translation of the β*-actin* mRNA. In particular, they proposed that ZBP1 associates with the β*-actin* transcript through the assembly of a localized mRNA-protein complex in the nucleus. Subsequently, ZBP1 mediates transport of the β*-actin* mRNA to the cytoplasm in a translationally repressed state (Hüttelmaier et al., 2005). This ZBP1-mediated inhibition of translation prevents premature protein synthesis. Translation can then occur when the ZBP1-RNA complex reaches its destination at the cell edge. Once at the periphery of the cell and in response to extracellular cues, ZBP1 can be phosphorylated by the protein kinase Src in a key tyrosine residue necessary for ZBP1’s RNA binding capacity (Hüttelmaier et al., 2005). This phosphorylation interferes with RNA binding and alleviates translational repression by decreasing ZBP1’s affinity to β*-actin* mRNA. Consequently, the β-actin mRNA can then be released and translated. The local increase in β-actin protein levels favors actin polymerization, cellular remodeling, and migration (Jung et al., 2014; Nicastro et al., 2017).

Another study by Welshhans and Bassell (2011), in ZBP1 deficient (ZBP1-/-) cortical neurons, demonstrates a genetic requirement for ZBP1 in local translation of β*-actin* and axon guidance. Especially following stimulation with cues like Netrin-1 and BDNF that elicit local mRNA translation and cytoskeletal rearrangements, the axonal growth cones of ZBP1-/- neurons exhibit attenuated localization of β*-actin* transcripts, as well as impaired β-actin local protein synthesis (Welshhans and Bassell, 2011). Furthermore, both filopodial dynamics and axon guidance are impaired in ZBP1-/- cortical neurons (Welshhans and Bassell, 2011). This is not a surprising consequence of ZBP1’s depletion (and subsequent β-actin translation impairment) since β-actin is mostly involved in filopodial dynamics (Suter and Forscher, 2000). Improper enrichment of β-actin protein in the growth cone is associated with impaired filopodial dynamics and axon guidance defects (Welshhans and Bassell, 2011).

Notably, ZBP1 has also been identified as a downstream mediator of non-canonical Sonic hedgehog (Shh) signaling during commissural axon guidance and providing the first link between Shh and growth cone cytoskeleton rearrangements (Lepelletier et al., 2017). Shh guides spinal cord commissural axons by attracting them toward the floorplate (Lepelletier et al., 2017). Local protein synthesis in response to Shh is the main driving force during this process. A study of Lepelletier et al. (2017) in rat commissural axons revealed that upon Shh stimulation, phospho-ZBP1 levels are increased in the growth cones. They also observed that Shh stimulation of axons that have been removed from the cell bodies results in increased β-actin protein levels in the growth cones (Lepelletier et al., 2017). On the other hand, depletion of ZBP1 *in vivo* results in commissural axon guidance defects (Lepelletier et al., 2017). Therefore, they suggest a model where stimulation of growth cones by Shh gradients induces ZBP1 phosphorylation and subsequent translation of its mRNA cargo, thereby allowing the growth cones to respond to Shh in a spatially defined manner (Lepelletier et al., 2017). Taking everything into account and considering the fact that ZBP1 can also bind to other mRNA molecules, such as the actin-related proteins (Arp) mRNAs which are involved in actin polymerization, ZBP1 seems to be a crucial factor for cytoskeleton dynamics by regulating local protein synthesis of specific cytoskeletal components and mediators (Jønson et al., 2007).

### The Shot-Kra complex

A study by Lee et al. (2007) highlights a new mechanism of how local mRNA translation can be coupled with cytoskeletal reorganization in the commissural neurons of *Drosophila melanogaster*. Short stop (Shot) is a neuronally expressed protein that constitutes a member of the cytoskeleton-associated plakin family in *D. melanogaster* (Lee and Kolodziej, 2002). Shot binds to, cross-links, and organizes both MTs and F-actin, thereby linking cytoskeletal structures together (Lee and Kolodziej, 2002; Sonnenberg and Liem, 2007). Krasavietz (KRA; also known as Extra bases) on the other hand is a novel Shot interactor identified in *D. melanogaster* (Lee et al., 2007). It contains a W2 motif which is also found in eIF5 and eIF2Bε, two translation initiation factors that regulate the activity of the heterotrimeric GTP-binding protein eIF2 (Lee et al., 2007). In its GTP-bound form, eIF2 is required for the recruitment of the initiator tRNA to the small (40S) ribosomal subunit (Howe and Hershey, 1984; Gavrilova et al., 1987). eIF2Bε and eIF5 regulate the activity of eIF2 by mediating GDP-GTP exchange and GTP hydrolysis (Chakrabarti and Maitra, 1991; Huang et al., 1997; Gomez et al., 2002; Kershaw et al., 2021).

Lee et al. (2007) conducted genetic complementation assays in *D. melanogaster* to highlight the crucial roles of Shot and Kra in midline axon guidance, a process highly dependent on Slit signaling via the Robo receptor. Based on their proposed model, Shot could serve as a cytoskeleton-localized platform for eIF2β and Kra, blocking eIF2β-mediated translation initiation in growth cones upon Slit-Robo repulsive signaling (Lee et al., 2007). They specifically found that Kra binds to the β-subunit of eIF2 through its W2 domain and associates with the growth cone cytoskeleton by physically interacting with Shot (Lee et al., 2007). *In vitro*, Kra inhibits global translation, suggesting a potential competition with eIF2Bε or eIF5 or both, for binding to eIF2β and blocking the initiation of translation by inhibiting the recruitment of the initiator tRNA to the 40S ribosomal subunit (Lee et al., 2007). Additionally, ectopic midline crossing defects due to loss of function mutations showed that eIF2β, as well as the W2 domain of Kra and the F-actin binding domain of Shot are crucial for proper midline axon guidance (Lee et al., 2007). Taken together, their study revealed that Slit-mediated midline repulsion requires the assembly of a functional, inhibitory, Shot-Kra-eIF2β translation complex that needs to be connected to the F-actin network to ensure proper function in the neuronal growth cone (Lee et al., 2007). Therefore, Shot can serve both as a cytoskeleton organizer and as a scaffold for translation regulators involved in midline axon guidance.

Although the Shot-Kra-eIF2β complex inhibits protein synthesis, it could have an alternative role in activating the translation of specific mRNAs. An interesting viewpoint regarding this potential additional function was described by Van Horck and Holt (2008). Based on the aforementioned model, Slit-induced repulsion would promote the translational activation of mRNAs that influence cytoskeletal disassembly, as well as the translational repression of mRNAs involved in cytoskeletal assembly. Thus, they hypothesized that the Shot-Kra-eIF2β complex could contribute to fine-tuning the balance between local mRNA translation repression and activation during midline axon guidance (Van Horck and Holt, 2008).

Interestingly, the mammalian homologs of Shot, namely, MACF1 and dystonin, are strongly expressed in the NS where they execute essential functions during development, as well as during maintenance/aging (Voelzmann et al., 2017). They interact with all cytoskeletal elements and affect important regulators of axonal MT, such as Tau and Map2. Although their function has not been studied with respect to mRNA translation, dystonin loss was reported to reduce levels of Tau and Map2 proteins, but not mRNAs, which could imply a potential role in translational control, alongside with their well-characterized function in cytoskeletal organization (Voelzmann et al., 2017).

## Concluding remarks

The highly polarized morphology and function of neurons is tightly bound to extremely complex, yet finely tuned intracellular processes, and precisely coordinated molecular mechanisms. Local mRNA translation, namely the ability of neurons to synthesize their proteins *in situ* and independently of the soma, is an astounding example of such sophisticated mechanisms. It enables remote axonal and dendritic subcellular compartments to remodel their proteome promptly and in response to local demand, allowing immediate responses to changes in the extracellular environment. It is not surprising therefore, that aberrant local translation in neuronal distal compartments has been correlated with numerous neurodevelopmental and neurodegenerative disorders, as well as the ability of axons to regenerate after injury. However, our current understanding of the mechanisms that regulate local mRNA translation is quite limited. On the other hand, the cytoskeleton is intrinsically linked to all aspects of NS development, maintenance, and function. Rapid rearrangements of cytoskeletal elements are required for neurons to be able to migrate, navigate their axons to synaptic targets, form and maintain synapses, and recover from traumatic injury. Therefore, it is not surprising that the regulation of the cytoskeleton has been extensively studied throughout the years, and numerous works have contributed to our understanding of filament formation, elongation, branching, and stability, as well as their implication to motility, guidance, synaptogenesis, and plasticity in the NS.

**FIGURE 1:**
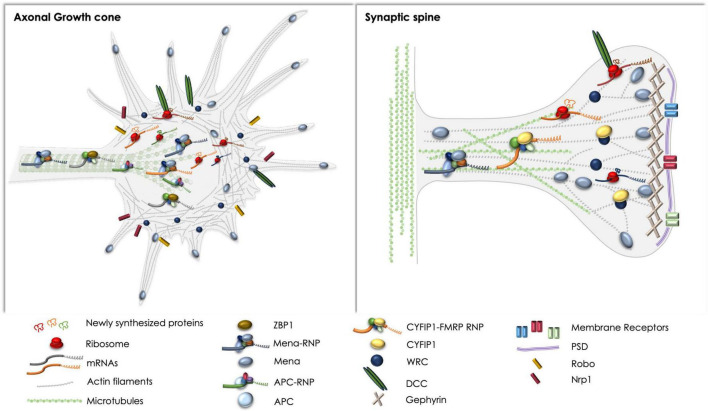
Schematic representation of the complexes discussed herein, with respect to basic components and spatial distribution in axonal growth cones (left) and synaptic spines (right). Note the regulatory complexes that have been reported and could potentially affect local protein synthesis and cytoskeletal rearrangements in both compartments, like the DCC receptor and the Mena-RNP. ZBP1 and the APC-RNP have been extensively studied in axons, whereas the CYFIP1-FMRP-RNP has been primarily examined in synapses. Potential crosstalk between the pathways remains elusive, although the direct or indirect association of receptors like DCC and Robo with Mena, or the WRC has been previously reported (Menzies et al., 2004; McConnell et al., 2016; Zou et al., 2018).

Given the innate connection of local mRNA translation and cytoskeletal reorganization, especially for prompt plastic responses to environmental stimuli, it is self-evident for neurons to retain common regulatory mechanisms for the sake of time and energy consumption. Yet, our current knowledge on those mechanisms and their coordination is very limited. A lot of effort has been put into uncovering specific mRNAs that are locally translated in different neuronal compartments, in an attempt to understand the molecular basis of axon development, synapse formation, and plasticity, as well as axonal response to injury. This has resulted in an extensive cataloging of transcripts that are specifically localized in subneuronal compartments, and are locally translated under different conditions. Notably, a large number of those transcripts encode cytoskeletal elements, like actin and tubulin, as well as cytoskeleton-associated proteins that bind to and stabilize cytoskeletal filaments. However, the proteins and protein complexes that regulate translation of those mRNAs in a precise spatiotemporal manner remain elusive, and so does our understanding of their crosstalk with the cytoskeleton. Herein, we have included protein complexes that appear to co-regulate mRNA translation and cytoskeletal remodeling, potentially connecting and balancing the two processes in the developing and adult NS (Figure 1). Nonetheless, a lot of research is still required, in order for us to fully apprehend the elegant means neurons possess to tune their molecular repertoires and intracellular procedures, in order to achieve prompt responses and precise function, both at the cellular and organismal level. Such knowledge could uncover novel therapeutic targets for neuronal disorders, and strategies based on the modulation of specific molecules with dual roles, tuning distinct processes toward the same outcome.

## References

[B1] Aakalu G. Smith W. B. Nguyen N. Jiang C. Schuman E. M. (2001). Dynamic visualization of local protein synthesis in hippocampal neurons. *Neuron* 30 489–502. 10.1016/s0896-6273(01)00295-1 11395009

[B2] Agrawal M. Welshhans K. (2021). Local translation across neural development: a focus on radial glial cells, axons, and synaptogenesis. *Front. Mol. Neurosci.* 14:717170. 10.3389/fnmol.2021.717170 34434089 PMC8380849

[B3] Alabed Y. Z. Grados-Munro E. Ferraro G. B. Hsieh S. H.-K. Fournier A. E. (2006). Neuronal responses to myelin are mediated by rho kinase. *J. Neurochem.* 96 1616–1625. 10.1111/j.1471-4159.2006.03670.x 16441511

[B4] Alonso M. Medina J. H. Pozzo-Miller L. (2004). ERK1/2 activation is necessary for BDNF to increase dendritic spine density in hippocampal CA1 pyramidal neurons. *Learn. Mem.* 11 172–178. 10.1101/lm.67804 15054132 PMC379687

[B5] Andreassi C. Luisier R. Crerar H. Darsinou M. Blokzijl-Franke S. Lenn T. (2021). Cytoplasmic cleavage of IMPA1 3’UTR is necessary for maintaining axon integrity. *Cell Rep.* 34:108778. 10.1016/j.celrep.2021.108778 33626357 PMC7918530

[B6] Applewhite D. A. Barzik M. Kojima S.-I. Svitkina T. M. Gertler F. B. Borisy G. G. (2007). Ena/VASP proteins have an anti-capping independent function in filopodia formation. *Mol. Biol. Cell* 18 2579–2591. 10.1091/mbc.e06-11-0990 17475772 PMC1924831

[B7] Attard T. M. Giglio P. Koppula S. Snyder C. Lynch H. T. (2007). Brain tumors in individuals with familial adenomatous polyposis: a cancer registry experience and pooled case report analysis. *Cancer* 109 761–766. 10.1002/cncr.22475 17238184

[B8] Autilio L. A. Appel S. H. Pettis P. Gambetti P. L. (1968). Biochemical studies of synapses *in vitro*. I. Protein synthesis. *Biochemistry* 7 2615–2622. 10.1021/bi00847a025 5660078

[B9] Baas P. W. Deitch J. S. Black M. M. Banker G. A. (1988). Polarity orientation of microtubules in hippocampal neurons: uniformity in the axon and nonuniformity in the dendrite. *Proc. Natl. Acad. Sci. U.S.A.* 85 8335–8339. 10.1073/pnas.85.21.8335 3054884 PMC282424

[B10] Bachmann C. Fischer L. Walter U. Reinhard M. (1999). The EVH2 domain of the vasodilator-stimulated phosphoprotein mediates tetramerization, F-actin binding, and actin bundle formation. *J. Biol. Chem.* 274 23549–23557. 10.1074/jbc.274.33.23549 10438535

[B11] Bagni C. Greenough W. T. (2005). From mRNP trafficking to spine dysmorphogenesis: the roots of fragile X syndrome. *Nat. Rev. Neurosci.* 6 376–387. 10.1038/nrn1667 15861180

[B12] Barnes A. P. Polleux F. (2009). Establishment of axon-dendrite polarity in developing neurons. *Annu. Rev. Neurosci.* 32 347–381. 10.1146/annurev.neuro.31.060407.125536 19400726 PMC3170863

[B13] Bartlett W. P. Banker G. A. (1984). An electron microscopic study of the development of axons and dendrites by hippocampal neurons in culture. II. Synaptic relationships. *J. Neurosci.* 4 1954–1965. 10.1523/JNEUROSCI.04-08-01954.1984 6470763 PMC6564956

[B14] Barzik M. Kotova T. I. Higgs H. N. Hazelwood L. Hanein D. Gertler F. B. (2005). Ena/VASP proteins enhance actin polymerization in the presence of barbed end capping proteins. *J. Biol. Chem.* 280 28653–28662. 10.1074/jbc.M503957200 15939738 PMC1747414

[B15] Bassell G. J. Warren S. T. (2008). Fragile X syndrome: loss of local mRNA regulation alters synaptic development and function. *Neuron* 60 201–214. 10.1016/j.neuron.2008.10.004 18957214 PMC3691995

[B16] Batista A. F. R. Hengst U. (2016). Intra-axonal protein synthesis in development and beyond. *Int. J. Dev. Neurosci.* 55 140–149. 10.1016/j.ijdevneu.2016.03.004 26970010 PMC5017888

[B17] Bear J. E. Gertler F. B. (2009). Ena/VASP: towards resolving a pointed controversy at the barbed end. *J. Cell Sci.* 122 1947–1953. 10.1242/jcs.038125 19494122 PMC2723151

[B18] Bear J. E. Svitkina T. M. Krause M. Schafer D. A. Loureiro J. J. Strasser G. A. (2002). Antagonism between Ena/VASP proteins and actin filament capping regulates fibroblast motility. *Cell* 109 509–521. 10.1016/s0092-8674(02)00731-6 12086607

[B19] Bellon A. Iyer A. Bridi S. Lee F. C. Y. Ovando-Vázquez C. Corradi E. (2017). miR-182 regulates Slit2-mediated axon guidance by modulating the local translation of a specific mRNA. *Cell Rep.* 18 1171–1186. 10.1016/j.celrep.2016.12.093 28147273 PMC5300892

[B20] Besse F. Ephrussi A. (2008). Translational control of localized mRNAs: restricting protein synthesis in space and time. *Nat. Rev. Mol. Cell Biol.* 9 971–980. 10.1038/nrm2548 19023284

[B21] Biever A. Donlin-Asp P. G. Schuman E. M. (2019). Local translation in neuronal processes. *Curr. Opin. Neurobiol.* 57 141–148. 10.1016/j.conb.2019.02.008 30861464

[B22] Bilancia C. G. Winkelman J. D. Tsygankov D. Nowotarski S. H. Sees J. A. Comber K. (2014). Enabled negatively regulates diaphanous-driven actin dynamics *in vitro* and *in vivo*. *Dev. Cell* 28 394–408. 10.1016/j.devcel.2014.01.015 24576424 PMC3992947

[B23] Borisoff J. F. Chan C. C. M. Hiebert G. W. Oschipok L. Robertson G. S. Zamboni R. (2003). Suppression of Rho-kinase activity promotes axonal growth on inhibitory CNS substrates. *Mol. Cell. Neurosci.* 22 405–416. 10.1016/s1044-7431(02)00032-5 12691741

[B24] Bosch M. Castro J. Saneyoshi T. Matsuno H. Sur M. Hayashi Y. (2014). Structural and molecular remodeling of dendritic spine substructures during long-term potentiation. *Neuron* 82 444–459. 10.1016/j.neuron.2014.03.021 24742465 PMC4281348

[B25] Bouchard J.-F. Horn K. E. Stroh T. Kennedy T. E. (2008). Depolarization recruits DCC to the plasma membrane of embryonic cortical neurons and enhances axon extension in response to netrin-1. *J. Neurochem.* 107 398–417. 10.1111/j.1471-4159.2008.05609.x 18691385

[B26] Boyer N. P. Gupton S. L. (2018). Revisiting Netrin-1: one who guides (Axons). *Front. Cell. Neurosci.* 12:221. 10.3389/fncel.2018.00221 30108487 PMC6080411

[B27] Breitsprecher D. Kiesewetter A. K. Linkner J. Vinzenz M. Stradal T. E. B. Small J. V. (2011). Molecular mechanism of Ena/VASP-mediated actin-filament elongation. *EMBO J.* 30 456–467. 10.1038/emboj.2010.348 21217643 PMC3034019

[B28] Broix L. Turchetto S. Nguyen L. (2021). Coordination between Transport and Local Translation in Neurons. *Trends Cell Biol*. 31 372–386. 10.1016/j.tcb.2021.01.001 33526339

[B29] Brown V. Jin P. Ceman S. Darnell J. C. O’Donnell W. T. Tenenbaum S. A. (2001). Microarray identification of FMRP-associated brain mRNAs and altered mRNA translational profiles in fragile X syndrome. *Cell* 107 477–487. 10.1016/s0092-8674(01)00568-2 11719188

[B30] Buxbaum A. R. Wu B. Signer R. H. (2014). Single β-actin mRNA detection in neurons reveals a mechanism for regulating its translatability. *Science* 343 419–422. 10.1126/science.1242939 24458642 PMC4121734

[B31] Cajigas I. J. Tushev G. Will T. J. tom Dieck S. Fuerst N. Schuman E. M. (2012). The local transcriptome in the synaptic neuropil revealed by deep sequencing and high-resolution imaging. *Neuron* 74 453–466. 10.1016/j.neuron.2012.02.036 22578497 PMC3627340

[B32] Cederquist G. Y. Luchniak A. Tischfield M. A. Peeva M. Song Y. Menezes M. P. (2012). An inherited TUBB2B mutation alters a kinesin-binding site and causes polymicrogyria, CFEOM and axon dysinnervation. *Hum. Mol. Genet.* 21 5484–5499. 10.1093/hmg/dds393 23001566 PMC3516133

[B33] Chakrabarti A. Maitra U. (1991). Function of eukaryotic initiation factor 5 in the formation of an 80 S ribosomal polypeptide chain initiation complex. *J. Biol. Chem.* 266 14039–14045. 1856230

[B34] Chen Z. Borek D. Padrick S. B. Gomez T. S. Metlagel Z. Ismail A. M. (2010). Structure and control of the actin regulatory WAVE complex. *Nature* 468 533–538. 10.1038/nature09623 21107423 PMC3085272

[B35] Chereau D. Dominguez R. (2006). Understanding the role of the G-actin-binding domain of Ena/VASP in actin assembly. *J. Struct. Biol.* 155 195–201. 10.1016/j.jsb.2006.01.012 16684607

[B36] Christie K. J. Webber C. A. Martinez J. A. Singh B. Zochodne D. W. (2010). PTEN inhibition to facilitate intrinsic regenerative outgrowth of adult peripheral axons. *J. Neurosci.* 30 9306–9315. 10.1523/JNEUROSCI.6271-09.2010 20610765 PMC6632469

[B37] Cioni J.-M. Koppers M. Holt C. E. (2018). Molecular control of local translation in axon development and maintenance. *Curr. Opin. Neurobiol.* 51 86–94. 10.1016/j.conb.2018.02.025 29549711

[B38] Cioni J.-M. Lin J. Q. Holtermann A. V. Koppers M. Jakobs M. A. H. Azizi A. (2019). Late endosomes act as mRNA translation platforms and sustain mitochondria in axons. *Cell* 176 56–72.e15 10.1016/j.cell.2018.11.030 30612743 PMC6333918

[B39] Clevers H. Nusse R. (2012). Wnt/β-catenin signaling and disease. *Cell* 149 1192–1205. 10.1016/j.cell.2012.05.012 22682243

[B40] Condeelis J. Singer R. H. (2005). How and why does beta-actin mRNA target? *Biol. Cell* 97 97–110. 10.1042/BC20040063 15601261

[B41] Conway A. E. Van Nostrand E. L. Pratt G. A. Aigner S. Wilbert M. L. Sundararaman B. (2016). Enhanced CLIP uncovers IMP Protein-RNA targets in human pluripotent stem cells important for cell adhesion and survival. *Cell Rep.* 15 666–679. 10.1016/j.celrep.2016.03.052 27068461 PMC4839292

[B42] Cooper G. M. Coe B. P. Girirajan S. Rosenfeld J. A. Vu T. H. Baker C. (2011). A copy number variation morbidity map of developmental delay. *Nat. Genet.* 43 838–846. 10.1038/ng.909 21841781 PMC3171215

[B43] Coutadeur S. Benyamine H. Delalonde L. de Oliveira C. Leblond B. Foucourt A. (2015). A novel DYRK1A (dual specificity tyrosine phosphorylation-regulated kinase 1A) inhibitor for the treatment of Alzheimer’s disease: effect on Tau and amyloid pathologies *in vitro*. *J. Neurochem.* 133 440–451. 10.1111/jnc.13018 25556849

[B44] Darnell J. C. Van Driesche S. J. Zhang C. Hung K. Y. S. Mele A. Fraser C. E. (2011). FMRP stalls ribosomal translocation on mRNAs linked to synaptic function and autism. *Cell* 146 247–261. 10.1016/j.cell.2011.06.013 21784246 PMC3232425

[B45] De Rubeis S. Pasciuto E. Li K. W. Fernández E. Di Marino D. Buzzi A. (2013). CYFIP1 coordinates mRNA translation and cytoskeleton remodeling to ensure proper dendritic spine formation. *Neuron* 79 1169–1182. 10.1016/j.neuron.2013.06.039 24050404 PMC3781321

[B46] Dent E. W. Gupton S. L. Gertler F. B. (2011). The growth cone cytoskeleton in axon outgrowth and guidance. *Cold Spring Harb. Perspect. Biol.* 3:a001800. 10.1101/cshperspect.a001800 21106647 PMC3039926

[B47] Dergham P. Ellezam B. Essagian C. Avedissian H. Lubell W. D. McKerracher L. (2002). Rho signaling pathway targeted to promote spinal cord repair. *J. Neurosci.* 22 6570–6577. 12151536 10.1523/JNEUROSCI.22-15-06570.2002 PMC6758168

[B48] Derisbourg M. Leghay C. Chiappetta G. Fernandez-Gomez F.-J. Laurent C. Demeyer D. (2015). Role of the Tau N-terminal region in microtubule stabilization revealed by new endogenous truncated forms. *Sci. Rep.* 5:9659. 10.1038/srep09659 25974414 PMC4431475

[B49] D’Este E. Kamin D. Göttfert F. El-Hady A. Hell S. W. (2015). STED nanoscopy reveals the ubiquity of subcortical cytoskeleton periodicity in living neurons. *Cell Rep.* 10 1246–1251. 10.1016/j.celrep.2015.02.007 25732815

[B50] Di Vona C. Bezdan D. Islam A. B. M. M. K. Salichs E. López-Bigas N. Ossowski S. (2015). Chromatin-wide profiling of DYRK1A reveals a role as a gene-specific RNA polymerase II CTD kinase. *Mol. Cell* 57 506–520. 10.1016/j.molcel.2014.12.026 25620562

[B51] Dickson B. J. (2002). Molecular mechanisms of axon guidance. *Science* 298 1959–1964. 10.1126/science.1072165 12471249

[B52] Dictenberg J. B. Swanger S. A. Antar L. N. Singer R. H. Bassell G. J. (2008). A direct role for FMRP in activity-dependent dendritic mRNA transport links filopodial-spine morphogenesis to fragile X syndrome. *Dev. Cell* 14 926–939. 10.1016/j.devcel.2008.04.003 18539120 PMC2453222

[B53] Donnelly C. J. Willis D. E. Xu M. Tep C. Jiang C. Yoo S. (2011). Limited availability of ZBP1 restricts axonal mRNA localization and nerve regeneration capacity. *EMBO J.* 30 4665–4677. 10.1038/emboj.2011.347 21964071 PMC3243598

[B54] Doornbos M. Sikkema-Raddatz B. Ruijvenkamp C. A. L. Dijkhuizen T. Bijlsma E. K. Gijsbers A. C. J. (2009). Nine patients with a microdeletion 15q11.2 between breakpoints 1 and 2 of the Prader-Willi critical region, possibly associated with behavioural disturbances. *Eur. J. Med. Genet.* 52 108–115. 10.1016/j.ejmg.2009.03.010 19328872

[B55] Drechsel D. N. Hyman A. A. Cobb M. H. Kirschner M. W. (1992). Modulation of the dynamic instability of tubulin assembly by the microtubule-associated protein tau. *Mol. Biol. Cell* 3 1141–1154. 10.1091/mbc.3.10.1141 1421571 PMC275678

[B56] Drees F. Gertler F. B. (2008). Ena/VASP: proteins at the tip of the nervous system. *Curr. Opin. Neurobiol.* 18 53–59. 10.1016/j.conb.2008.05.007 18508258 PMC2515615

[B57] Dwivedy A. Gertler F. B. Miller J. Holt C. E. Lebrand C. (2007). Ena/VASP function in retinal axons is required for terminal arborization but not pathway navigation. *Development* 134 2137–2146. 10.1242/dev.002345 17507414 PMC3792372

[B58] Eden S. Rohatgi R. Podtelejnikov A. V. Mann M. Kirschner M. W. (2002). Mechanism of regulation of WAVE1-induced actin nucleation by Rac1 and Nck. *Nature* 418 790–793. 10.1038/nature00859 12181570

[B59] Ehninger D. Silva A. J. (2009). Genetics and neuropsychiatric disorders: treatment during adulthood. *Nat. Med.* 15 849–850. 10.1038/nm0809-849 19661989

[B60] Eom T. Antar L. N. Singer R. H. Bassell G. J. (2003). Localization of a beta-actin messenger ribonucleoprotein complex with zipcode-binding protein modulates the density of dendritic filopodia and filopodial synapses. *J. Neurosci.* 23 10433–10444. 10.1523/JNEUROSCI.23-32-10433.2003 14614102 PMC6741001

[B61] Erskine L. Herrera E. (2007). The retinal ganglion cell axon’s journey: insights into molecular mechanisms of axon guidance. *Dev. Biol.* 308 1–14. 10.1016/j.ydbio.2007.05.013 17560562

[B62] Ertürk A. Hellal F. Enes J. Bradke F. (2007). Disorganized microtubules underlie the formation of retraction bulbs and the failure of axonal regeneration. *J. Neurosci.* 27 9169–9180. 10.1523/JNEUROSCI.0612-07.2007 17715353 PMC6672197

[B63] Etienne-Manneville S. Hall A. (2003). Cdc42 regulates GSK-3beta and adenomatous polyposis coli to control cell polarity. *Nature* 421 753–756. 10.1038/nature01423 12610628

[B64] Etienne-Manneville S. Manneville J.-B. Nicholls S. Ferenczi M. A. Hall A. (2005). Cdc42 and Par6-PKCzeta regulate the spatially localized association of Dlg1 and APC to control cell polarization. *J. Cell Biol.* 170 895–901. 10.1083/jcb.200412172 16157700 PMC2171429

[B65] Farina K. L. Huttelmaier S. Musunuru K. Darnell R. Singer R. H. (2003). Two ZBP1 KH domains facilitate beta-actin mRNA localization, granule formation, and cytoskeletal attachment. *J. Cell Biol.* 160 77–87. 10.1083/jcb.200206003 12507992 PMC2172732

[B66] Fazeli A. Dickinson S. L. Hermiston M. L. Tighe R. V. Steen R. G. Small C. G. (1997). Phenotype of mice lacking functional Deleted in colorectal cancer (Dcc) gene. *Nature* 386 796–804. 10.1038/386796a0 9126737

[B67] Fehlings M. G. Theodore N. Harrop J. Maurais G. Kuntz C. Shaffrey C. I. (2011). A phase I/IIa clinical trial of a recombinant Rho protein antagonist in acute spinal cord injury. *J. Neurotrauma* 28 787–796. 10.1089/neu.2011.1765 21381984

[B68] Ferron F. Rebowski G. Lee S. H. Dominguez R. (2007). Structural basis for the recruitment of profilin-actin complexes during filament elongation by Ena/VASP. *EMBO J.* 26 4597–4606. 10.1038/sj.emboj.7601874 17914456 PMC2063483

[B69] Finci L. I. Zhang J. Sun X. Smock R. G. Meijers R. Zhang Y. (2017). Structure of unliganded membrane-proximal domains FN4-FN5-FN6 of DCC. *Protein Cell* 8 701–705. 10.1007/s13238-017-0439-x 28664251 PMC5563286

[B70] Flynn K. C. Hellal F. Neukirchen D. Jacob S. Tahirovic S. Dupraz S. (2012). ADF/cofilin-mediated actin retrograde flow directs neurite formation in the developing brain. *Neuron* 76 1091–1107. 10.1016/j.neuron.2012.09.038 23259946

[B71] Folci A. Mapelli L. Sassone J. Prestori F. D’Angelo E. Bassani S. (2014). Loss of hnRNP K impairs synaptic plasticity in hippocampal neurons. *J. Neurosci.* 34 9088–9095. 10.1523/JNEUROSCI.0303-14.2014 24990929 PMC6608249

[B72] Forrest K. M. Gavis E. R. (2003). Live imaging of endogenous RNA reveals a diffusion and entrapment mechanism for nanos mRNA localization in *Drosophila*. *Curr. Biol.* 13 1159–1168. 10.1016/s0960-9822(03)00451-2 12867026

[B73] Fournier A. E. Takizawa B. T. Strittmatter S. M. (2003). Rho kinase inhibition enhances axonal regeneration in the injured CNS. *J. Neurosci.* 23 1416–1423. 12598630 10.1523/JNEUROSCI.23-04-01416.2003 PMC6742251

[B74] Gasparski A. N. Mason D. E. Moissoglu K. Mili S. (2022). Regulation and outcomes of localized RNA translation. *Wiley Interdiscip. Rev. RNA.* [Epub ahead of print]. 10.1002/wrna.1721 35166036 PMC9787767

[B75] Gates J. Mahaffey J. P. Rogers S. L. Emerson M. Rogers E. M. Sottile S. L. (2007). Enabled plays key roles in embryonic epithelial morphogenesis in *Drosophila*. *Development* 134 2027–2039. 10.1242/dev.02849 17507404

[B76] Gavrilova L. P. Rutkevitch N. M. Gelfand V. I. Motuz L. P. Stahl J. Bommer U. A. (1987). Immunofluorescent localization of protein synthesis components in mouse embryo fibroblasts. *Cell Biol. Int. Rep.* 11 745–753. 10.1016/0309-1651(87)90134-2 3319194

[B77] Gebauer F. Hentze M. W. (2004). Molecular mechanisms of translational control. *Nat. Rev. Mol. Cell Biol.* 5 827–835. 10.1038/nrm1488 15459663 PMC7097087

[B78] Giesemann T. Schwarz G. Nawrotzki R. Berhörster K. Rothkegel M. Schlüter K. (2003). Complex formation between the postsynaptic scaffolding protein gephyrin, profilin, and Mena: a possible link to the microfilament system. *J. Neurosci.* 23 8330–8339. 10.1523/JNEUROSCI.23-23-08330.2003 12967995 PMC6740687

[B79] Giger R. J. Hollis E. R. Tuszynski M. H. (2010). Guidance molecules in axon regeneration. *Cold Spring Harb. Perspect. Biol.* 2:a001867. 10.1101/cshperspect.a001867 20519341 PMC2890195

[B80] Gkogkas C. G. Khoutorsky A. Ran I. Rampakakis E. Nevarko T. Weatherill D. B. (2013). Autism-related deficits via dysregulated eIF4E-dependent translational control. *Nature* 493 371–377. 10.1038/nature11628 23172145 PMC4133997

[B81] Glock C. Biever A. Tushev G. Nassim-Assir B. Kao A. Bartnik I. (2021). The translatome of neuronal cell bodies, dendrites, and axons. *Proc. Natl. Acad. Sci. U.S.A.* 118:e2113929118. 10.1073/pnas.2113929118 34670838 PMC8639352

[B82] Gomes R. A. Hampton C. El-Sabeawy F. Sabo S. L. McAllister A. K. (2006). The dynamic distribution of TrkB receptors before, during, and after synapse formation between cortical neurons. *J. Neurosci.* 26 11487–11500. 10.1523/JNEUROSCI.2364-06.2006 17079678 PMC6674530

[B83] Gomez E. Mohammad S. S. Pavitt G. D. (2002). Characterization of the minimal catalytic domain within eIF2B: the guanine-nucleotide exchange factor for translation initiation. *EMBO J.* 21 5292–5301. 10.1093/emboj/cdf515 12356745 PMC129037

[B84] Gordon-Weeks P. R. Fournier A. E. (2014). Neuronal cytoskeleton in synaptic plasticity and regeneration. *J. Neurochem.* 129 206–212. 10.1111/jnc.12502 24147810

[B85] Grantham J. Ruddock L. W. Roobol A. Carden M. J. (2002). Eukaryotic chaperonin containing T-complex polypeptide 1 interacts with filamentous actin and reduces the initial rate of actin polymerization *in vitro*. *Cell Stress Chaperones* 7 235–242. 10.1379/1466-1268(2002)007<0235:ecctcp>2.0.co;2 12482199 PMC514823

[B86] Green R. A. Kaplan K. B. (2003). Chromosome instability in colorectal tumor cells is associated with defects in microtubule plus-end attachments caused by a dominant mutation in APC. *J. Cell Biol.* 163 949–961. 10.1083/jcb.200307070 14662741 PMC2173599

[B87] Grevengoed E. E. Peifer M. (2003). Cytoskeletal connections: building strong cells in new ways. *Curr. Biol.* 13 R568–R570. 10.1016/s0960-9822(03)00476-7 12867052

[B88] Grove M. Demyanenko G. Echarri A. Zipfel P. A. Quiroz M. E. Rodriguiz R. M. (2004). ABI2-deficient mice exhibit defective cell migration, aberrant dendritic spine morphogenesis, and deficits in learning and memory. *Mol. Cell. Biol.* 24 10905–10922. 10.1128/MCB.24.24.10905-10922.2004 15572692 PMC533973

[B89] Grutzendler J. Kasthuri N. Gan W.-B. (2002). Long-term dendritic spine stability in the adult cortex. *Nature* 420 812–816. 10.1038/nature01276 12490949

[B90] Gumy L. F. Tan C. L. Fawcett J. W. (2010). The role of local protein synthesis and degradation in axon regeneration. *Exp. Neurol.* 223 28–37. 10.1016/j.expneurol.2009.06.004 19520073 PMC2864402

[B91] Gupton S. L. Gertler F. B. (2010). Integrin signaling switches the cytoskeletal and exocytic machinery that drives neuritogenesis. *Dev. Cell* 18 725–736. 10.1016/j.devcel.2010.02.017 20493807 PMC3383070

[B92] Gupton S. L. Riquelme D. Hughes-Alford S. K. Tadros J. Rudina S. S. Hynes R. O. (2012). Mena binds α5 integrin directly and modulates α5β1 function. *J. Cell Biol.* 198 657–676. 10.1083/jcb.201202079 22908313 PMC3514034

[B93] Hafner A.-S. Donlin-Asp P. G. Leitch B. Herzog E. Schuman E. M. (2019). Local protein synthesis is a ubiquitous feature of neuronal pre- and postsynaptic compartments. *Science* 364:eaau3644. 10.1126/science.aau3644 31097639

[B94] Hansen S. D. Mullins R. D. (2010). VASP is a processive actin polymerase that requires monomeric actin for barbed end association. *J. Cell Biol.* 191 571–584. 10.1083/jcb.201003014 21041447 PMC3003327

[B95] Hanz S. Perlson E. Willis D. Zheng J.-Q. Massarwa R. Huerta J. J. (2003). Axoplasmic importins enable retrograde injury signaling in lesioned nerve. *Neuron* 40 1095–1104. 10.1016/s0896-6273(03)00770-0 14687545

[B96] Harker A. J. Katkar H. H. Bidone T. C. Aydin F. Voth G. A. Applewhite D. A. (2019). Ena/VASP processive elongation is modulated by avidity on actin filaments bundled by the filopodia cross-linker fascin. *Mol. Biol. Cell* 30 851–862. 10.1091/mbc.E18-08-0500 30601697 PMC6589784

[B97] Harris W. A. Holt C. E. Bonhoeffer F. (1987). Retinal axons with and without their somata, growing to and arborizing in the tectum of Xenopus embryos: a time-lapse video study of single fibres *in vivo*. *Development* 101 123–133. 10.1242/dev.101.1.123 3449363

[B98] Harvey C. D. Yasuda R. Zhong H. Svoboda K. (2008). The spread of Ras activity triggered by activation of a single dendritic spine. *Science* 321 136–140. 10.1126/science.1159675 18556515 PMC2745709

[B99] Hatton D. D. Sideris J. Skinner M. Mankowski J. Bailey D. B. Roberts J. (2006). Autistic behavior in children with fragile X syndrome: prevalence, stability, and the impact of FMRP. *Am. J. Med. Genet. A* 140A 1804–1813. 10.1002/ajmg.a.31286 16700053

[B100] Havrylenko S. Noguera P. Abou-Ghali M. Manzi J. Faqir F. Lamora A. (2015). WAVE binds Ena/VASP for enhanced Arp2/3 complex-based actin assembly. *Mol. Biol. Cell* 26 55–65. 10.1091/mbc.E14-07-1200 25355952 PMC4279229

[B101] Hayashi-Takagi A. Yagishita S. Nakamura M. Shirai F. Wu Y. I. Loshbaugh A. L. (2015). Labelling and optical erasure of synaptic memory traces in the motor cortex. *Nature* 525 333–338. 10.1038/nature15257 26352471 PMC4634641

[B102] Hedrick N. G. Harward S. C. Hall C. E. Murakoshi H. McNamara J. O. Yasuda R. (2016). Rho GTPase complementation underlies BDNF-dependent homo- and heterosynaptic plasticity. *Nature* 538 104–108. 10.1038/nature19784 27680697 PMC5361895

[B103] Hirokawa N. Niwa S. Tanaka Y. (2010). Molecular motors in neurons: transport mechanisms and roles in brain function, development, and disease. *Neuron* 68 610–638. 10.1016/j.neuron.2010.09.039 21092854

[B104] Holt C. E. Schuman E. M. (2013). The central dogma decentralized: new perspectives on RNA function and local translation in neurons. *Neuron* 80 648–657. 10.1016/j.neuron.2013.10.036 24183017 PMC3820025

[B105] Horn K. E. Glasgow S. D. Gobert D. Bull S.-J. Luk T. Girgis J. (2013). DCC expression by neurons regulates synaptic plasticity in the adult brain. *Cell Rep.* 3 173–185. 10.1016/j.celrep.2012.12.005 23291093

[B106] Hotulainen P. Hoogenraad C. C. (2010). Actin in dendritic spines: connecting dynamics to function. *J. Cell Biol.* 189 619–629. 10.1083/jcb.201003008 20457765 PMC2872912

[B107] Hou L. Antion M. D. Hu D. Spencer C. M. Paylor R. Klann E. (2006). Dynamic translational and proteasomal regulation of fragile X mental retardation protein controls mGluR-dependent long-term depression. *Neuron* 51 441–454. 10.1016/j.neuron.2006.07.005 16908410

[B108] Howe J. G. Hershey J. W. (1984). Translational initiation factor and ribosome association with the cytoskeletal framework fraction from HeLa cells. *Cell* 37 85–93. 10.1016/0092-8674(84)90303-9 6722878

[B109] Huang H. K. Yoon H. Hannig E. M. Donahue T. F. (1997). GTP hydrolysis controls stringent selection of the AUG start codon during translation initiation in Saccharomyces cerevisiae. *Genes Dev*. 11 2396–2413. 10.1101/gad.11.18.2396 9308967 PMC316512

[B110] Hüttelmaier S. Zenklusen D. Lederer M. Dictenberg J. Lorenz M. Meng X. (2005). Spatial regulation of beta-actin translation by Src-dependent phosphorylation of ZBP1. *Nature* 438 512–515. 10.1038/nature04115 16306994

[B111] Irwin S. A. Idupulapati M. Gilbert M. E. Harris J. B. Chakravarti A. B. Rogers E. J. (2002). Dendritic spine and dendritic field characteristics of layer V pyramidal neurons in the visual cortex of fragile-X knockout mice. *Am. J. Med. Genet.* 111 140–146. 10.1002/ajmg.10500 12210340

[B112] Jacquemont S. Hagerman R. J. Hagerman P. J. Leehey M. A. (2007). Fragile-X syndrome and fragile X-associated tremor/ataxia syndrome: two faces of FMR1. *Lancet Neurol.* 6 45–55. 10.1016/S1474-4422(06)70676-7 17166801

[B113] Jaglin X. H. Poirier K. Saillour Y. Buhler E. Tian G. Bahi-Buisson N. (2009). Mutations in the beta-tubulin gene TUBB2B result in asymmetrical polymicrogyria. *Nat. Genet.* 41 746–752. 10.1038/ng.380 19465910 PMC2883584

[B114] Janke C. (2014). The tubulin code: molecular components, readout mechanisms, and functions. *J. Cell Biol.* 206 461–472. 10.1083/jcb.201406055 25135932 PMC4137062

[B115] Jaworski J. Kapitein L. C. Gouveia S. M. Dortland B. R. Wulf P. S. Grigoriev I. (2009). Dynamic microtubules regulate dendritic spine morphology and synaptic plasticity. *Neuron* 61 85–100. 10.1016/j.neuron.2008.11.013 19146815

[B116] Ji S.-J. Jaffrey S. R. (2012). Intra-axonal translation of SMAD1/5/8 mediates retrograde regulation of trigeminal ganglia subtype specification. *Neuron* 74 95–107. 10.1016/j.neuron.2012.02.022 22500633 PMC3328135

[B117] Jones K. J. Korb E. Kundel M. A. Kochanek A. R. Kabraji S. McEvoy M. (2008). CPEB1 regulates beta-catenin mRNA translation and cell migration in astrocytes. *Glia* 56 1401–1413. 10.1002/glia.20707 18618654 PMC3013359

[B118] Jønson L. Vikesaa J. Krogh A. Nielsen L. K. Hansen T. V. Borup R. (2007). Molecular composition of IMP1 ribonucleoprotein granules. *Mol. Cell. Proteomics* 6 798–811. 10.1074/mcp.M600346-MCP200 17289661

[B119] Jovanovic J. N. Czernik A. J. Fienberg A. A. Greengard P. Sihra T. S. (2000). Synapsins as mediators of BDNF-enhanced neurotransmitter release. *Nat. Neurosci.* 3 323–329. 10.1038/73888 10725920

[B120] Jung H. Gkogkas C. G. Sonenberg N. Holt C. E. (2014). Remote control of gene function by local translation. *Cell* 157 26–40. 10.1016/j.cell.2014.03.005 24679524 PMC3988848

[B121] Jung H. Yoon B. C. Holt C. E. (2012). Axonal mRNA localization and local protein synthesis in nervous system assembly, maintenance and repair. *Nat. Rev. Neurosci.* 13 308–324. 10.1038/nrn3210 22498899 PMC3682205

[B122] Kalinski A. L. Sachdeva R. Gomes C. Lee S. J. Shah Z. Houle J. D. (2015). mRNAs and protein synthetic machinery localize into regenerating spinal cord axons when they are provided a substrate that supports growth. *J. Neurosci.* 35 10357–10370. 10.1523/JNEUROSCI.1249-15.2015 26180210 PMC4502271

[B123] Kalkman H. O. (2012). A review of the evidence for the canonical Wnt pathway in autism spectrum disorders. *Mol. Autism* 3:10. 10.1186/2040-2392-3-10 23083465 PMC3492093

[B124] Kang H. Schuman E. M. (1995). Long-lasting neurotrophin-induced enhancement of synaptic transmission in the adult hippocampus. *Science* 267 1658–1662. 10.1126/science.7886457 7886457

[B125] Kang H. Welcher A. A. Shelton D. Schuman E. M. (1997). Neurotrophins and time: different roles for TrkB signaling in hippocampal long-term potentiation. *Neuron* 19 653–664. 10.1016/s0896-6273(00)80378-5 9331355

[B126] Kapitein L. C. Schlager M. A. Kuijpers M. Wulf P. S. van Spronsen M. MacKintosh F. C. (2010). Mixed microtubules steer dynein-driven cargo transport into dendrites. *Curr. Biol.* 20 290–299. 10.1016/j.cub.2009.12.052 20137950

[B127] Kawasaki Y. Sato R. Akiyama T. (2003). Mutated APC and Asef are involved in the migration of colorectal tumour cells. *Nat. Cell Biol.* 5 211–215. 10.1038/ncb937 12598901

[B128] Keino-Masu K. Masu M. Hinck L. Leonardo E. D. Chan S. S. Culotti J. G. (1996). Deleted in Colorectal Cancer (DCC) encodes a netrin receptor. *Cell* 87 175–185. 10.1016/s0092-8674(00)81336-7 8861902

[B129] Kershaw C. J. Jennings M. D. Cortopassi F. Guaita M. Al-Ghafli H. Pavitt G. D. (2021). GTP binding to translation factor eIF2B stimulates its guanine nucleotide exchange activity. *iScience* 24:103454. 10.1016/j.isci.2021.103454 34877508 PMC8633983

[B130] Ketschek A. Jones S. Spillane M. Korobova F. Svitkina T. Gallo G. (2015). Nerve growth factor promotes reorganization of the axonal microtubule array at sites of axon collateral branching. *Dev. Neurobiol.* 75 1441–1461. 10.1002/dneu.22294 25846486 PMC4827620

[B131] Kevenaar J. T. Hoogenraad C. C. (2015). The axonal cytoskeleton: from organization to function. *Front. Mol. Neurosci.* 8:44. 10.3389/fnmol.2015.00044 26321907 PMC4536388

[B132] Kim S. Coulombe P. A. (2010). Emerging role for the cytoskeleton as an organizer and regulator of translation. *Nat. Rev. Mol. Cell Biol.* 11 75–81. 10.1038/nrm2818 20027187

[B133] Kim Y. Sung J. Y. Ceglia I. Lee K.-W. Ahn J.-H. Halford J. M. (2006). Phosphorylation of WAVE1 regulates actin polymerization and dendritic spine morphology. *Nature* 442 814–817. 10.1038/nature04976 16862120

[B134] Kita K. Wittmann T. Näthke I. S. Waterman-Storer C. M. (2006). Adenomatous polyposis coli on microtubule plus ends in cell extensions can promote microtubule net growth with or without EB1. *Mol. Biol. Cell* 17 2331–2345. 10.1091/mbc.e05-06-0498 16525027 PMC1446093

[B135] Klemmer P. Meredith R. M. Holmgren C. D. Klychnikov O. I. Stahl-Zeng J. Loos M. (2011). Proteomics, ultrastructure, and physiology of hippocampal synapses in a fragile X syndrome mouse model reveal presynaptic phenotype. *J. Biol. Chem.* 286 25495–25504. 10.1074/jbc.M110.210260 21596744 PMC3138307

[B136] Kobayashi K. Kuroda S. Fukata M. Nakamura T. Nagase T. Nomura N. (1998). p140Sra-1 (specifically Rac1-associated protein) is a novel specific target for Rac1 small GTPase. *J. Biol. Chem.* 273 291–295. 10.1074/jbc.273.1.291 9417078

[B137] Koenig E. (1967). Synthetic mechanisms in the axon. IV. *In vitro* incorporation of [3H]precursors into axonal protein and RNA. *J. Neurochem.* 14 437–446. 10.1111/j.1471-4159.1967.tb09542.x 5336968

[B138] Koenig E. Martin R. Titmus M. Sotelo-Silveira J. R. (2000). Cryptic peripheral ribosomal domains distributed intermittently along mammalian myelinated axons. *J. Neurosci.* 20 8390–8400. 10.1523/JNEUROSCI.20-22-08390.2000 11069946 PMC6773183

[B139] Koester M. P. Müller O. Pollerberg G. E. (2007). Adenomatous polyposis coli is differentially distributed in growth cones and modulates their steering. *J. Neurosci.* 27 12590–12600. 10.1523/JNEUROSCI.2250-07.2007 18003838 PMC6673337

[B140] Kolodkin A. L. Tessier-Lavigne M. (2011). Mechanisms and molecules of neuronal wiring: a primer. *Cold Spring Harb. Perspect. Biol.* 3:a001727. 10.1101/cshperspect.a001727 21123392 PMC3098670

[B141] Kolodziej P. A. Timpe L. C. Mitchell K. J. Fried S. R. Goodman C. S. Jan L. Y. (1996). frazzled encodes a Drosophila member of the DCC immunoglobulin subfamily and is required for CNS and motor axon guidance. *Cell* 87 197–204. 10.1016/s0092-8674(00)81338-0 8861904

[B142] Kroboth K. Newton I. P. Kita K. Dikovskaya D. Zumbrunn J. Waterman-Storer C. M. (2007). Lack of adenomatous polyposis coli protein correlates with a decrease in cell migration and overall changes in microtubule stability. *Mol. Biol. Cell* 18 910–918. 10.1091/mbc.e06-03-0179 17192415 PMC1805109

[B143] Krumm N. O’Roak B. J. Shendure J. Eichler E. E. (2014). A de novo convergence of autism genetics and molecular neuroscience. *Trends Neurosci.* 37 95–105. 10.1016/j.tins.2013.11.005 24387789 PMC4077788

[B144] Kunda P. Craig G. Dominguez V. Baum B. (2003). Abi, Sra1, and Kette control the stability and localization of SCAR/WAVE to regulate the formation of actin-based protrusions. *Curr. Biol.* 13 1867–1875. 10.1016/j.cub.2003.10.005 14588242

[B145] Kwiatkowski A. V. Rubinson D. A. Dent E. W. Edward van Veen J. Leslie J. D. Zhang J. (2007). Ena/VASP Is Required for neuritogenesis in the developing cortex. *Neuron* 56 441–455. 10.1016/j.neuron.2007.09.008 17988629

[B146] Lang S. B. Stein V. Bonhoeffer T. Lohmann C. (2007). Endogenous brain-derived neurotrophic factor triggers fast calcium transients at synapses in developing dendrites. *J. Neurosci.* 27 1097–1105. 10.1523/JNEUROSCI.3590-06.2007 17267564 PMC6673203

[B147] Lanier L. M. Gates M. A. Witke W. Menzies A. S. Wehman A. M. Macklis J. D. (1999). Mena is required for neurulation and commissure formation. *Neuron* 22 313–325. 10.1016/s0896-6273(00)81092-2 10069337

[B148] Lawrence J. B. Singer R. H. (1986). Intracellular localization of messenger RNAs for cytoskeletal proteins. *Cell* 45 407–415. 10.1016/0092-8674(86)90326-0 3698103

[B149] Lebrand C. Dent E. W. Strasser G. A. Lanier L. M. Krause M. Svitkina T. M. (2004). Critical role of Ena/VASP proteins for filopodia formation in neurons and in function downstream of netrin-1. *Neuron* 42 37–49. 10.1016/s0896-6273(04)00108-4 15066263

[B150] Lee S. Kolodziej P. A. (2002). Short Stop provides an essential link between F-actin and microtubules during axon extension. *Development* 129 1195–1204. 10.1242/dev.129.5.1195 11874915

[B151] Lee S. Nahm M. Lee M. Kwon M. Kim E. Zadeh A. D. (2007). The F-actin-microtubule crosslinker Shot is a platform for Krasavietz-mediated translational regulation of midline axon repulsion. *Development* 134 1767–1777. 10.1242/dev.02842 17409115

[B152] Leeds P. Kren B. T. Boylan J. M. Betz N. A. Steer C. J. Gruppuso P. A. (1997). Developmental regulation of CRD-BP, an RNA-binding protein that stabilizes c-myc mRNA *in vitro*. *Oncogene* 14 1279–1286. 10.1038/sj.onc.1201093 9178888

[B153] Lepelletier L. Langlois S. D. Kent C. B. Welshhans K. Morin S. Bassell G. J. (2017). Sonic hedgehog guides axons via zipcode binding protein 1-mediated local translation. *J. Neurosci.* 37 1685–1695. 10.1523/JNEUROSCI.3016-16.2016 28073938 PMC6589976

[B154] Letourneau P. C. (2009). Actin in axons: stable scaffolds and dynamic filaments. *Results Probl. Cell Differ.* 48 65–90. 10.1007/400_2009_15 19582412

[B155] Leung K.-M. Lu B. Wong H. H.-W. Lin J. Q. Turner-Bridger B. Holt C. E. (2018). Cue-polarized transport of β-actin mRNA Depends on 3’UTR and microtubules in live growth cones. *Front. Cell. Neurosci.* 12:300. 10.3389/fncel.2018.00300 30250426 PMC6139529

[B156] Leung K.-M. van Horck F. P. G. Lin A. C. Allison R. Standart N. Holt C. E. (2006). Asymmetrical beta-actin mRNA translation in growth cones mediates attractive turning to netrin-1. *Nat. Neurosci.* 9 1247–1256. 10.1038/nn1775 16980963 PMC1997306

[B157] Leung L. C. Urbanèiè V. Baudet M.-L. Dwivedy A. Bayley T. G. Lee A. C. (2013). Coupling of NF-protocadherin signaling to axon guidance by cue-induced translation. *Nat. Neurosci.* 16 166–173. 10.1038/nn.3290 23292679 PMC3701881

[B158] Levine E. S. Dreyfus C. F. Black I. B. Plummer M. R. (1995). Brain-derived neurotrophic factor rapidly enhances synaptic transmission in hippocampal neurons via postsynaptic tyrosine kinase receptors. *Proc. Natl. Acad. Sci. U.S.A.* 92 8074–8077. 10.1073/pnas.92.17.8074 7644540 PMC41289

[B159] Li W. Li Y. Gao F.-B. (2005). Abelson, enabled, and p120 catenin exert distinct effects on dendritic morphogenesis in *Drosophila*. *Dev. Dyn.* 234 512–522. 10.1002/dvdy.20496 16003769

[B160] Li W. Ma L. Yang G. Gan W.-B. (2017). REM sleep selectively prunes and maintains new synapses in development and learning. *Nat. Neurosci.* 20 427–437. 10.1038/nn.4479 28092659 PMC5535798

[B161] Liao L. Park S. K. Xu T. Vanderklish P. Yates J. R. (2008). Quantitative proteomic analysis of primary neurons reveals diverse changes in synaptic protein content in fmr1 knockout mice. *Proc. Natl. Acad. Sci. U.S.A.* 105 15281–15286. 10.1073/pnas.0804678105 18829439 PMC2563066

[B162] Lin Y.-L. Lei Y.-T. Hong C.-J. Hsueh Y.-P. (2007). Syndecan-2 induces filopodia and dendritic spine formation via the neurofibromin-PKA-Ena/VASP pathway. *J. Cell Biol.* 177 829–841. 10.1083/jcb.200608121 17548511 PMC2064283

[B163] Lu R. Wang H. Liang Z. Ku L. O’donnell W. T. Li W. (2004). The fragile X protein controls microtubule-associated protein 1B translation and microtubule stability in brain neuron development. *Proc. Natl. Acad. Sci. U.S.A.* 101 15201–15206. 10.1073/pnas.0404995101 15475576 PMC524058

[B164] Lukinavièius G. Reymond L. D’Este E. Masharina A. Göttfert F. Ta H. (2014). Fluorogenic probes for live-cell imaging of the cytoskeleton. *Nat. Methods* 11 731–733. 10.1038/nmeth.2972 24859753

[B165] Luo L. (2002). Actin cytoskeleton regulation in neuronal morphogenesis and structural plasticity. *Annu. Rev. Cell Dev. Biol.* 18 601–635. 10.1146/annurev.cellbio.18.031802.150501 12142283

[B166] Manitt C. Mimee A. Eng C. Pokinko M. Stroh T. Cooper H. M. (2011). The netrin receptor DCC is required in the pubertal organization of mesocortical dopamine circuitry. *J. Neurosci.* 31 8381–8394. 10.1523/JNEUROSCI.0606-11.2011 21653843 PMC6623341

[B167] McConnell R. E. Edward van Veen J. Vidaki M. Kwiatkowski A. V. Meyer A. S. Gertler F. B. (2016). A requirement for filopodia extension toward Slit during Robo-mediated axon repulsion. *J. Cell Biol.* 213 261–274. 10.1083/jcb.201509062 27091449 PMC5084274

[B168] McKerracher L. Higuchi H. (2006). Targeting Rho to stimulate repair after spinal cord injury. *J. Neurotrauma* 23 309–317. 10.1089/neu.2006.23.309 16629618

[B169] Medioni C. Ramialison M. Ephrussi A. Besse F. (2014). Imp promotes axonal remodeling by regulating profilin mRNA during brain development. *Curr. Biol.* 24 793–800. 10.1016/j.cub.2014.02.038 24656828

[B170] Menzies A. S. Aszodi A. Williams S. E. Pfeifer A. Wehman A. M. Goh K. L. (2004). Mena and vasodilator-stimulated phosphoprotein are required for multiple actin-dependent processes that shape the vertebrate nervous system. *J. Neurosci.* 24 8029–8038. 10.1523/JNEUROSCI.1057-04.2004 15371503 PMC6729793

[B171] Merriam E. B. Lumbard D. C. Viesselmann C. Ballweg J. Stevenson M. Pietila L. (2011). Dynamic microtubules promote synaptic NMDA receptor-dependent spine enlargement. *PLoS One* 6:e27688. 10.1371/journal.pone.0027688 22096612 PMC3214068

[B172] Merriam E. B. Millette M. Lumbard D. C. Saengsawang W. Fothergill T. Hu X. (2013). Synaptic regulation of microtubule dynamics in dendritic spines by calcium, F-actin, and drebrin. *J. Neurosci.* 33 16471–16482. 10.1523/JNEUROSCI.0661-13.2013 24133252 PMC3797370

[B173] Mili S. Moissoglu K. Macara I. G. (2008). Genome-Wide Screen Identifies Localized RNAs Anchored At Cell Protrusions Through Microtubules And APC. *Nature* 453 115–119. 10.1038/nature06888 18451862 PMC2782773

[B174] Mimori-Kiyosue Y. Shiina N. Tsukita S. (2000). Adenomatous polyposis coli (APC) protein moves along microtubules and concentrates at their growing ends in epithelial cells. *J. Cell Biol.* 148 505–518. 10.1083/jcb.148.3.505 10662776 PMC2174811

[B175] Mitchison T. Kirschner M. (1984). Dynamic instability of microtubule growth. *Nature* 312 237–242. 10.1038/312237a0 6504138

[B176] Miyashiro K. Y. Beckel-Mitchener A. Purk T. P. Becker K. G. Barret T. Liu L. (2003). RNA cargoes associating with FMRP reveal deficits in cellular functioning in Fmr1 null mice. *Neuron* 37 417–431. 10.1016/s0896-6273(03)00034-5 12575950

[B177] Mofatteh M. (2020). mRNA localization and local translation in neurons. *AIMS Neurosci*. 7 299–310. 10.3934/Neuroscience.2020016 32995487 PMC7519968

[B178] Mohajerani M. H. Sivakumaran S. Zacchi P. Aguilera P. Cherubini E. (2007). Correlated network activity enhances synaptic efficacy via BDNF and the ERK pathway at immature CA3 CA1 connections in the hippocampus. *Proc. Natl. Acad. Sci. U.S.A.* 104 13176–13181. 10.1073/pnas.0704533104 17656555 PMC1941828

[B179] Morgan I. G. Austin L. (1968). Synaptosomal protein synthesis in a cell-free system. *J. Neurochem.* 15 41–51. 10.1111/j.1471-4159.1968.tb06172.x 4230127

[B180] Murakoshi H. Wang H. Yasuda R. (2011). Local, persistent activation of Rho GTPases during plasticity of single dendritic spines. *Nature* 472 100–104. 10.1038/nature09823 21423166 PMC3105377

[B181] Nakahata Y. Yasuda R. (2018). Plasticity of spine structure: local signaling, translation and cytoskeletal reorganization. *Front. Synaptic Neurosci.* 10:29. 10.3389/fnsyn.2018.00029 30210329 PMC6123351

[B182] Napoli I. Mercaldo V. Boyl P. P. Eleuteri B. Zalfa F. De Rubeis S. (2008). The fragile X syndrome protein represses activity-dependent translation through CYFIP1, a new 4E-BP. *Cell* 134 1042–1054. 10.1016/j.cell.2008.07.031 18805096

[B183] Näthke I. S. Adams C. L. Polakis P. Sellin J. H. Nelson W. J. (1996). The adenomatous polyposis coli tumor suppressor protein localizes to plasma membrane sites involved in active cell migration. *J. Cell Biol.* 134 165–179. 10.1083/jcb.134.1.165 8698812 PMC2120913

[B184] Neves-Pereira M. Müller B. Massie D. Williams J. H. G. O’Brien P. C. M. Hughes A. (2009). Deregulation of EIF4E: a novel mechanism for autism. *J. Med. Genet.* 46 759–765. 10.1136/jmg.2009.066852 19556253

[B185] Nicastro G. Candel A. M. Uhl M. Oregioni A. Hollingworth D. Backofen R. (2017). Mechanism of β-actin mRNA Recognition by ZBP1. *Cell Rep.* 18 1187–1199. 10.1016/j.celrep.2016.12.091 28147274 PMC5300891

[B186] Nielsen J. Christiansen J. Lykke-Andersen J. Johnsen A. H. Wewer U. M. Nielsen F. C. (1999). A family of insulin-like growth factor II mRNA-binding proteins represses translation in late development. *Mol. Cell. Biol.* 19 1262–1270. 10.1128/MCB.19.2.1262 9891060 PMC116055

[B187] Nishino J. Kim S. Zhu Y. Zhu H. Morrison S. J. (2013). A network of heterochronic genes including Imp1 regulates temporal changes in stem cell properties. *eLife* 2:e00924. 10.7554/eLife.00924 24192035 PMC3817382

[B188] Nowicki S. T. Tassone F. Ono M. Y. Ferranti J. Croquette M. F. Goodlin-Jones B. (2007). The Prader-Willi phenotype of fragile X syndrome. *J. Dev. Behav. Pediatr.* 28 133–138. 10.1097/01.DBP.0000267563.18952.c9 17435464

[B189] O’Roak B. J. Vives L. Fu W. Egertson J. D. Stanaway I. B. Phelps I. G. (2012). Multiplex targeted sequencing identifies recurrently mutated genes in autism spectrum disorders. *Science* 338 1619–1622. 10.1126/science.1227764 23160955 PMC3528801

[B190] Ostroff L. E. Cain C. K. Jindal N. Dar N. Ledoux J. E. (2012). Stability of presynaptic vesicle pools and changes in synapse morphology in the amygdala following fear learning in adult rats. *J. Comp. Neurol.* 520 295–314. 10.1002/cne.22691 21674493 PMC3590817

[B191] Ostroff L. E. Watson D. J. Cao G. Parker P. H. Smith H. Harris K. M. (2018). Shifting patterns of polyribosome accumulation at synapses over the course of hippocampal long-term potentiation. *Hippocampus* 28 416–430. 10.1002/hipo.22841 29575288 PMC5992065

[B192] Parent A. T. Barnes N. Y. Taniguchi Y. Thinakaran G. Sisodia S. S. (2005). Presenilin attenuates receptor-mediated signaling and synaptic function. *J. Neurosci.* 25 1540–1549. 10.1523/JNEUROSCI.3850-04.2005 15703408 PMC6725985

[B193] Patel V. L. Mitra S. Harris R. Buxbaum A. R. Lionnet T. Brenowitz M. (2012). Spatial arrangement of an RNA zipcode identifies mRNAs under post-transcriptional control. *Genes Dev.* 26 43–53. 10.1101/gad.177428.111 22215810 PMC3258965

[B194] Penzes P. Cahill M. E. Jones K. A. VanLeeuwen J.-E. Woolfrey K. M. (2011). Dendritic spine pathology in neuropsychiatric disorders. *Nat. Neurosci.* 14 285–293. 10.1038/nn.2741 21346746 PMC3530413

[B195] Pinto-Costa R. Sousa S. C. Leite S. C. Nogueira-Rodrigues J. Ferreira da Silva T. Machado D. (2020). Profilin 1 delivery tunes cytoskeletal dynamics toward CNS axon regeneration. *J. Clin. Invest.* 130 2024–2040. 10.1172/JCI125771 31945017 PMC7108904

[B196] Piper M. Anderson R. Dwivedy A. Weinl C. van Horck F. Leung K. M. (2006). Signaling mechanisms underlying Slit2-induced collapse of Xenopus retinal growth cones. *Neuron* 49 215–228. 10.1016/j.neuron.2005.12.008 16423696 PMC3689199

[B197] Piper M. Lee A. C. van Horck F. P. G. McNeilly H. Lu T. B. Harris W. A. (2015). Differential requirement of F-actin and microtubule cytoskeleton in cue-induced local protein synthesis in axonal growth cones. *Neural Dev*. 10:3. 10.1186/s13064-015-0031-0 25886013 PMC4350973

[B198] Powell S. M. Zilz N. Beazer-Barclay Y. Bryan T. M. Hamilton S. R. Thibodeau S. N. (1992). APC mutations occur early during colorectal tumorigenesis. *Nature* 359 235–237. 10.1038/359235a0 1528264

[B199] Pradhan J. Noakes P. G. Bellingham M. C. (2019). The role of Altered BDNF/TrkB signaling in amyotrophic lateral sclerosis. *Front. Cell. Neurosci.* 13:368. 10.3389/fncel.2019.00368 31456666 PMC6700252

[B200] Preitner N. Quan J. Nowakowski D. W. Hancock M. L. Shi J. Tcherkezian J. (2014). APC is an RNA-binding protein and its interactome provides a link to neural development and microtubule assembly. *Cell* 158 368–382. 10.1016/j.cell.2014.05.042 25036633 PMC4133101

[B201] Purro S. A. Ciani L. Hoyos-Flight M. Stamatakou E. Siomou E. Salinas P. C. (2008). Wnt regulates axon behavior through changes in microtubule growth directionality: a new role for adenomatous polyposis coli. *J. Neurosci.* 28 8644–8654. 10.1523/JNEUROSCI.2320-08.2008 18716223 PMC2832753

[B202] Qian W. Jin N. Shi J. Yin X. Jin X. Wang S. (2013). Dual-specificity tyrosine phosphorylation-regulated kinase 1A (Dyrk1A) enhances tau expression. *J. Alzheimers Dis.* 37 529–538. 10.3233/JAD-130824 23948904

[B203] Rangaraju V. Lauterbach M. Schuman E. M. (2019). Spatially stable mitochondrial compartments fuel local translation during plasticity. *Cell* 176 73–84.e15. 10.1016/j.cell.2018.12.013 30612742

[B204] Reyes S. Fu Y. Double K. L. Cottam V. Thompson L. H. Kirik D. (2013). Trophic factors differentiate dopamine neurons vulnerable to Parkinson’s disease. *Neurobiol. Aging* 34 873–886. 10.1016/j.neurobiolaging.2012.07.019 22926168

[B205] Romaniello R. Tonelli A. Arrigoni F. Baschirotto C. Triulzi F. Bresolin N. (2012). A novel mutation in the β-tubulin gene TUBB2B associated with complex malformation of cortical development and deficits in axonal guidance. *Dev. Med. Child Neurol.* 54 765–769. 10.1111/j.1469-8749.2012.04316.x 22591407

[B206] Rubinfeld B. Souza B. Albert I. Müller O. Chamberlain S. H. Masiarz F. R. (1993). Association of the APC gene product with beta-catenin. *Science* 262 1731–1734. 10.1126/science.8259518 8259518

[B207] Russell S. A. Bashaw G. J. (2018). Axon guidance pathways and the control of gene expression. *Dev. Dyn.* 247 571–580. 10.1002/dvdy.24609 29226467 PMC6167058

[B208] Sanchez G. Dury A. Y. Murray L. M. Biondi O. Tadesse H. El Fatimy R. (2013). A novel function for the survival motoneuron protein as a translational regulator. *Hum. Mol. Genet.* 22 668–684. 10.1093/hmg/dds474 23136128

[B209] Santini E. Huynh T. N. Longo F. Koo S. Y. Mojica E. D’Andrea L. (2017). Reducing eIF4E-eIF4G interactions restores the balance between protein synthesis and actin dynamics in fragile X syndrome model mice. *Sci. Signal.* 10:eaan0665. 10.1126/scisignal.aan0665 29114037 PMC5858943

[B210] Santini E. Huynh T. N. MacAskill A. F. Carter A. G. Pierre P. Ruggero D. (2013). Exaggerated translation causes synaptic and behavioural aberrations associated with autism. *Nature* 493 411–415. 10.1038/nature11782 23263185 PMC3548017

[B211] Sasaki Y. Welshhans K. Wen Z. Yao J. Xu M. Goshima Y. (2010). Phosphorylation of zipcode binding protein 1 is required for brain-derived neurotrophic factor signaling of local beta-actin synthesis and growth cone turning. *J. Neurosci.* 30 9349–9358. 10.1523/JNEUROSCI.0499-10.2010 20631164 PMC2908896

[B212] Saxton R. A. Sabatini D. M. (2017). mTOR signaling in growth, metabolism, and disease. *Cell* 169 361–371. 10.1016/j.cell.2017.03.035 28388417

[B213] Scarnati M. S. Kataria R. Biswas M. Paradiso K. G. (2018). Active presynaptic ribosomes in the mammalian brain, and altered transmitter release after protein synthesis inhibition. *eLife* 7:e36697. 10.7554/eLife.36697 30375975 PMC6231766

[B214] Schenck A. Bardoni B. Langmann C. Harden N. Mandel J. L. Giangrande A. (2003). CYFIP/Sra-1 controls neuronal connectivity in *Drosophila* and links the Rac1 GTPase pathway to the fragile X protein. *Neuron* 38 887–898. 10.1016/s0896-6273(03)00354-4 12818175

[B215] Schenck A. Bardoni B. Moro A. Bagni C. Mandel J. L. (2001). A highly conserved protein family interacting with the fragile X mental retardation protein (FMRP) and displaying selective interactions with FMRP-related proteins FXR1P and FXR2P. *Proc. Natl. Acad. Sci. U.S.A.* 98 8844–8849. 10.1073/pnas.151231598 11438699 PMC37523

[B216] Schratt G. M. Nigh E. A. Chen W. G. Hu L. Greenberg M. E. (2004). BDNF regulates the translation of a select group of mRNAs by a mammalian target of rapamycin-phosphatidylinositol 3-kinase-dependent pathway during neuronal development. *J. Neurosci.* 24 7366–7377. 10.1523/JNEUROSCI.1739-04.2004 15317862 PMC6729778

[B217] Segal R. A. Greenberg M. E. (1996). Intracellular signaling pathways activated by neurotrophic factors. *Annu. Rev. Neurosci.* 19 463–489. 10.1146/annurev.ne.19.030196.002335 8833451

[B218] Shi S.-H. Cheng T. Jan L. Y. Jan Y.-N. (2004). APC and GSK-3beta are involved in mPar3 targeting to the nascent axon and establishment of neuronal polarity. *Curr. Biol.* 14 2025–2032. 10.1016/j.cub.2004.11.009 15556865

[B219] Sieber O. M. Heinimann K. Gorman P. Lamlum H. Crabtree M. Simpson C. A. (2002). Analysis of chromosomal instability in human colorectal adenomas with two mutational hits at APC. *Proc. Natl. Acad. Sci. U.S.A.* 99 16910–16915. 10.1073/pnas.012679099 12486240 PMC139243

[B220] Skruber K. Warp P. V. Shklyarov R. Thomas J. D. Swanson M. S. Henty-Ridilla J. L. (2020). Arp2/3 and Mena/VASP require profilin 1 for actin network assembly at the leading edge. *Curr. Biol.* 30 2651–2664.e5. 10.1016/j.cub.2020.04.085 32470361 PMC7375932

[B221] Soderling S. H. Guire E. S. Kaech S. White J. Zhang F. Schutz K. (2007). A WAVE-1 and WRP signaling complex regulates spine density, synaptic plasticity, and memory. *J. Neurosci.* 27 355–365. 10.1523/JNEUROSCI.3209-06.2006 17215396 PMC3740594

[B222] Song M. Martinowich K. Lee F. S. (2017). BDNF at the synapse: why location matters. *Mol. Psychiatry* 22 1370–1375. 10.1038/mp.2017.144 28937692 PMC5646361

[B223] Sonnenberg A. Liem R. K. H. (2007). Plakins in development and disease. *Exp. Cell Res.* 313 2189–2203. 10.1016/j.yexcr.2007.03.039 17499243

[B224] Spillane M. Ketschek A. Donnelly C. J. Pacheco A. Twiss J. L. Gallo G. (2012). Nerve growth factor-induced formation of axonal filopodia and collateral branches involves the intra-axonal synthesis of regulators of the actin-nucleating Arp2/3 complex. *J. Neurosci.* 32 17671–17689. 10.1523/JNEUROSCI.1079-12.2012 23223289 PMC3596264

[B225] Spillane M. Ketschek A. Merianda T. T. Twiss J. L. Gallo G. (2013). Mitochondria coordinate sites of axon branching through localized intra-axonal protein synthesis. *Cell Rep.* 5 1564–1575. 10.1016/j.celrep.2013.11.022 24332852 PMC3947524

[B226] Stepanova T. Slemmer J. Hoogenraad C. C. Lansbergen G. Dortland B. De Zeeuw C. I. (2003). Visualization of microtubule growth in cultured neurons via the use of EB3-GFP (end-binding protein 3-green fluorescent protein). *J. Neurosci.* 23 2655–2664. 10.1523/JNEUROSCI.23-07-02655.2003 12684451 PMC6742099

[B227] Steward O. Levy W. B. (1982). Preferential localization of polyribosomes under the base of dendritic spines in granule cells of the dentate gyrus. *J. Neurosci.* 2 284–291. 10.1523/JNEUROSCI.02-03-00284.1982 7062109 PMC6564334

[B228] Steward O. Schuman E. M. (2003). Compartmentalized synthesis and degradation of proteins in neurons. *Neuron* 40 347–359. 10.1016/s0896-6273(03)00635-4 14556713

[B229] Stone M. C. Roegiers F. Rolls M. M. (2008). Microtubules have opposite orientation in axons and dendrites of *Drosophila* neurons. *Mol. Biol. Cell* 19 4122–4129. 10.1091/mbc.e07-10-1079 18667536 PMC2555934

[B230] Suter D. M. Forscher P. (2000). Substrate-cytoskeletal coupling as a mechanism for the regulation of growth cone motility and guidance. *J. Neurobiol.* 44 97–113. 10934315

[B231] Svitkina T. M. Bulanova E. A. Chaga O. Y. Vignjevic D. M. Kojima S. Vasiliev J. M. (2003). Mechanism of filopodia initiation by reorganization of a dendritic network. *J. Cell Biol.* 160 409–421. 10.1083/jcb.200210174 12566431 PMC2172658

[B232] Takenawa T. Suetsugu S. (2007). The WASP-WAVE protein network: connecting the membrane to the cytoskeleton. *Nat. Rev. Mol. Cell Biol.* 8 37–48. 10.1038/nrm2069 17183359

[B233] Tam G. W. C. van de Lagemaat L. N. Redon R. Strathdee K. E. Croning M. D. R. Malloy M. P. (2010). Confirmed rare copy number variants implicate novel genes in schizophrenia. *Biochem. Soc. Trans.* 38 445–451. 10.1042/BST0380445 20298200

[B234] Tanaka E. Sabry J. (1995). Making the connection: cytoskeletal rearrangements during growth cone guidance. *Cell* 83 171–176. 10.1016/0092-8674(95)90158-2 7585934

[B235] Tcherkezian J. Brittis P. A. Thomas F. Roux P. P. Flanagan J. G. (2021). Transmembrane receptor DCC associates with protein synthesis machinery and regulates translation. *Cell* 184:2520. 10.1016/j.cell.2021.04.018 33930296 PMC8189100

[B236] Tejedor F. J. Hämmerle B. (2011). MNB/DYRK1A as a multiple regulator of neuronal development. *FEBS J*. 278 223–235. 10.1111/j.1742-4658.2010.07954.x 21156027

[B237] Torres-Berrio A. Hernandez G. Nestler E. J. Flores C. (2020). The Netrin-1/DCC guidance cue pathway as a molecular target in depression: translational evidence. *Biol. Psychiatry* 88 611–624. 10.1016/j.biopsych.2020.04.025 32593422 PMC7529861

[B238] Tortosa E. Galjart N. Avila J. Sayas C. L. (2013). MAP1B regulates microtubule dynamics by sequestering EB1/3 in the cytosol of developing neuronal cells. *EMBO J.* 32 1293–1306. 10.1038/emboj.2013.76 23572079 PMC3642684

[B239] Trachtenberg J. T. Chen B. E. Knott G. W. Feng G. Sanes J. R. Welker E. (2002). Long-term *in vivo* imaging of experience-dependent synaptic plasticity in adult cortex. *Nature* 420 788–794. 10.1038/nature01273 12490942

[B240] Tucker P. K. Evans I. R. Wood W. (2011). Ena drives invasive macrophage migration in *Drosophila* embryos. *Dis. Model. Mech.* 4 126–134. 10.1242/dmm.005694 21045209 PMC3008967

[B241] Ueberham U. Thomas A. (2013). “The role of SMAD proteins for development, differentiation and dedifferentiation of neurons,” in *Trends in Cell Signaling Pathways in Neuronal Fate Decision*, ed. Wislet-Gendebien S. (Rijeka: IntechOpen), 75–111. 10.5772/54532

[B242] Vale R. D. (2003). The molecular motor toolbox for intracellular transport. *Cell* 112 467–480. 10.1016/s0092-8674(03)00111-9 12600311

[B243] van Bon B. W. M. Coe B. P. Bernier R. Green C. Gerdts J. Witherspoon K. (2016). Disruptive de novo mutations of DYRK1A lead to a syndromic form of autism and ID. *Mol. Psychiatry* 21 126–132. 10.1038/mp.2015.5 25707398 PMC4547916

[B244] van der Zwaag B. Staal W. G. Hochstenbach R. Poot M. Spierenburg H. A. de Jonge M. V. (2010). A co-segregating microduplication of chromosome 15q11.2 pinpoints two risk genes for autism spectrum disorder. *Am. J. Med. Genet. B Neuropsychiatr. Genet.* 153B 960–966. 10.1002/ajmg.b.31055 20029941 PMC2933514

[B245] Van Horck F. P. G. Holt C. E. (2008). A cytoskeletal platform for local translation in axons. *Sci. Signal.* 1:e11. 10.1126/stke.18pe11 18314505 PMC3682639

[B246] Venticinque L. Jamieson K. V. Meruelo D. (2011). Interactions between laminin receptor and the cytoskeleton during translation and cell motility. *PLoS One* 6:e15895. 10.1371/journal.pone.0015895 21249134 PMC3017552

[B247] Verma P. Chierzi S. Codd A. M. Campbell D. S. Meyer R. L. Holt C. E. (2005). Axonal protein synthesis and degradation are necessary for efficient growth cone regeneration. *J. Neurosci.* 25 331–342. 10.1523/JNEUROSCI.3073-04.2005 15647476 PMC3687202

[B248] Vidaki M. Drees F. Saxena T. Lanslots E. Taliaferro M. J. Tatarakis A. (2017). A requirement for Mena, an actin regulator, in local mRNA translation in developing neurons. *Neuron* 95 608–622.e5. 10.1016/j.neuron.2017.06.048 28735747 PMC5616167

[B249] Vitriol E. A. Zheng J. Q. (2012). Growth cone travel in space and time: the cellular ensemble of cytoskeleton, adhesion, and membrane. *Neuron* 73 1068–1081. 10.1016/j.neuron.2012.03.005 22445336 PMC3319373

[B250] Voelzmann A. Liew Y.-T. Qu Y. Hahn I. Melero C. Sánchez-Soriano N. (2017). Drosophila Short stop as a paradigm for the role and regulation of spectraplakins. *Semin. Cell Dev. Biol.* 69 40–57. 10.1016/j.semcdb.2017.05.019 28579450

[B251] von der Lippe C. Rustad C. Heimdal K. Rødningen O. K. (2011). 15q11.2 microdeletion - seven new patients with delayed development and/or behavioural problems. *Eur. J. Med. Genet.* 54 357–360. 10.1016/j.ejmg.2010.12.008 21187176

[B252] Wahl S. Barth H. Ciossek T. Aktories K. Mueller B. K. (2000). Ephrin-A5 induces collapse of growth cones by activating Rho and Rho kinase. *J. Cell Biol.* 149 263–270. 10.1083/jcb.149.2.263 10769020 PMC2175154

[B253] Walders-Harbeck B. Khaitlina S. Y. Hinssen H. Jockusch B. M. Illenberger S. (2002). The vasodilator-stimulated phosphoprotein promotes actin polymerisation through direct binding to monomeric actin. *FEBS Lett.* 529 275–280. 10.1016/s0014-5793(02)03356-2 12372613

[B254] Wang C. S. Kavalali E. T. Monteggia L. M. (2022). BDNF signaling in context: from synaptic regulation to psychiatric disorders. *Cell* 185 62–76. 10.1016/j.cell.2021.12.003 34963057 PMC8741740

[B255] Watanabe T. Wang S. Noritake J. Sato K. Fukata M. Takefuji M. (2004). Interaction with IQGAP1 links APC to Rac1, Cdc42, and actin filaments during cell polarization and migration. *Dev. Cell* 7 871–883. 10.1016/j.devcel.2004.10.017 15572129

[B256] Weidensdorfer D. Stöhr N. Baude A. Lederer M. Köhn M. Schierhorn A. (2009). Control of c-myc mRNA stability by IGF2BP1-associated cytoplasmic RNPs. *RNA* 15 104–115. 10.1261/rna.1175909 19029303 PMC2612774

[B257] Weil T. T. Parton R. Davis I. Gavis E. R. (2008). Changes in bicoid mRNA anchoring highlight conserved mechanisms during the oocyte-to-embryo transition. *Curr. Biol.* 18 1055–1061. 10.1016/j.cub.2008.06.046 18639459 PMC2581475

[B258] Welshhans K. Bassell G. J. (2011). Netrin-1-induced local β-actin synthesis and growth cone guidance requires zipcode binding protein 1. *J. Neurosci.* 31 9800–9813. 10.1523/JNEUROSCI.0166-11.2011 21734271 PMC3137872

[B259] Wen Y. Eng C. H. Schmoranzer J. Cabrera-Poch N. Morris E. J. S. Chen M. (2004). EB1 and APC bind to mDia to stabilize microtubules downstream of Rho and promote cell migration. *Nat. Cell Biol.* 6 820–830. 10.1038/ncb1160 15311282

[B260] Westmark C. J. Malter J. S. (2007). FMRP mediates mGluR5-dependent translation of amyloid precursor protein. *PLoS Biol.* 5:e52. 10.1371/journal.pbio.0050052 17298186 PMC1808499

[B261] Wiens K. M. Lin H. Liao D. (2005). Rac1 induces the clustering of AMPA receptors during spinogenesis. *J. Neurosci.* 25 10627–10636. 10.1523/JNEUROSCI.1947-05.2005 16291935 PMC6725855

[B262] Winkelman J. D. Bilancia C. G. Peifer M. Kovar D. R. (2014). Ena/VASP Enabled is a highly processive actin polymerase tailored to self-assemble parallel-bundled F-actin networks with Fascin. *Proc. Natl. Acad. Sci. U.S.A.* 111 4121–4126. 10.1073/pnas.1322093111 24591594 PMC3964058

[B263] Xu K. Zhong G. Zhuang X. (2013). Actin, spectrin, and associated proteins form a periodic cytoskeletal structure in axons. *Science* 339 452–456. 10.1126/science.1232251 23239625 PMC3815867

[B264] Yang G. Pan F. Gan W.-B. (2009). Stably maintained dendritic spines are associated with lifelong memories. *Nature* 462 920–924. 10.1038/nature08577 19946265 PMC4724802

[B265] Yao J. Sasaki Y. Wen Z. Bassell G. J. Zheng J. Q. (2006). An essential role for beta-actin mRNA localization and translation in Ca2+-dependent growth cone guidance. *Nat. Neurosci.* 9 1265–1273. 10.1038/nn1773 16980965

[B266] Yasuda K. Zhang H. Loiselle D. Haystead T. Macara I. G. Mili S. (2013). The RNA-binding protein Fus directs translation of localized mRNAs in APC-RNP granules. *J. Cell Biol.* 203 737–746. 10.1083/jcb.201306058 24297750 PMC3857475

[B267] Yin Y. Edelman G. M. Vanderklish P. W. (2002). The brain-derived neurotrophic factor enhances synthesis of Arc in synaptoneurosomes. *Proc. Natl. Acad. Sci. U.S.A.* 99 2368–2373. 10.1073/pnas.042693699 11842217 PMC122371

[B268] Yisraeli J. K. (2005). VICKZ proteins: a multi-talented family of regulatory RNA-binding proteins. *Biol. Cell* 97 87–96. 10.1042/BC20040151 15601260

[B269] Yuan A. Rao M. V. Veeranna Nixon R. A. (2017). Neurofilaments and neurofilament proteins in health and disease. *Cold Spring Harb. Perspect. Biol* 9:a018309. 10.1101/cshperspect.a018309 28373358 PMC5378049

[B270] Yukawa K. Tanaka T. Bai T. Ueyama T. Owada-Makabe K. Tsubota Y. (2005). Semaphorin 4A induces growth cone collapse of hippocampal neurons in a Rho/Rho-kinase-dependent manner. *Int. J. Mol. Med.* 16 115–118. 15942687

[B271] Zalfa F. Eleuteri B. Dickson K. S. Mercaldo V. De Rubeis S. di Penta A. (2007). A new function for the fragile X mental retardation protein in regulation of PSD-95 mRNA stability. *Nat. Neurosci.* 10 578–587. 10.1038/nn1893 17417632 PMC2804293

[B272] Zalfa F. Giorgi M. Primerano B. Moro A. Di Penta A. Reis S. (2003). The fragile X syndrome protein FMRP associates with BC1 RNA and regulates the translation of specific mRNAs at synapses. *Cell* 112 317–327. 10.1016/s0092-8674(03)00079-5 12581522

[B273] Zappulo A. van den Bruck D. Ciolli Mattioli C. Franke V. Imami K. McShane E. (2017). RNA localization is a key determinant of neurite-enriched proteome. *Nat. Commun.* 8:583. 10.1038/s41467-017-00690-6 28928394 PMC5605627

[B274] Zeng X. Tamai K. Doble B. Li S. Huang H. Habas R. (2005). A dual-kinase mechanism for Wnt co-receptor phosphorylation and activation. *Nature* 438 873–877. 10.1038/nature04185 16341017 PMC2100418

[B275] Zhang M. Wang Q. Huang Y. (2007). Fragile X mental retardation protein FMRP and the RNA export factor NXF2 associate with and destabilize Nxf1 mRNA in neuronal cells. *Proc. Natl. Acad. Sci. U.S.A.* 104 10057–10062. 10.1073/pnas.0700169104 17548835 PMC1891223

[B276] Zhang Y. Q. Bailey A. M. Matthies H. J. Renden R. B. Smith M. A. Speese S. D. (2001). Drosophila fragile X-related gene regulates the MAP1B homolog Futsch to control synaptic structure and function. *Cell* 107 591–603. 10.1016/s0092-8674(01)00589-x 11733059

[B277] Zhao Q. Li T. Zhao X. Huang K. Wang T. Li Z. (2013). Rare CNVs and tag SNPs at 15q11.2 are associated with schizophrenia in the Han Chinese population. *Schizophr. Bull.* 39 712–719. 10.1093/schbul/sbr197 22317777 PMC3627771

[B278] Zimyanin V. L. Belaya K. Pecreaux J. Gilchrist M. J. Clark A. Davis I. (2008). *In vivo* imaging of oskar mRNA transport reveals the mechanism of posterior localization. *Cell* 134 843–853. 10.1016/j.cell.2008.06.053 18775316 PMC2585615

[B279] Zivraj K. H. Tung Y. C. L. Piper M. Gumy L. Fawcett J. W. Yeo G. S. H. (2010). Subcellular profiling reveals distinct and developmentally regulated repertoire of growth cone mRNAs. *J. Neurosci.* 30 15464–15478. 10.1523/JNEUROSCI.1800-10.2010 21084603 PMC3683943

[B280] Zou W. Dong X. Broederdorf T. R. Shen A. Kramer D. A. Shi R. (2018). A dendritic guidance receptor complex brings together distinct actin regulators to drive efficient f-actin assembly and branching. *Dev. Cell* 45 362–375.e3. 10.1016/j.devcel.2018.04.008 29738713 PMC6292511

